# Supplementary material for: Neutralizing the pathological effects of extracellular histones with small polyanions
Source: Nat Commun. 2020 Dec 16;11:6408. doi: 10.1038/s41467-020-20231-y (PMC7744542; doi:10.1038/s41467-020-20231-y)
Supplement: Supplementary file 1 — Supplementary Information [file 41467_2020_20231_MOESM1_ESM.pdf]

SUPPLEMENTARY INFORMATION

Neutralizing the pathological effects of extracellular histones with small polyanions

Connor H. O'Meara,[1,8] Lucy A. Coupland,[1,8] Farzaneh Kordbacheh,[1,8] Benjamin J. C. Quah,[1,8] Chih-Wei Chang,[2,8] David A. Simon Davis,[1] Anna Bezos,[1] Anna M. Browne,[1] Craig Freeman,[1] Dillon J. Hammill,[1] Pradeep Chopra,[2] Gergely Pipa,[2] Paul D. Madge,[2] Esther Gallant,[3] Courtney Segovis,[3] Angela F. Dulhunty,[3] Leonard F. Arnolda,[4] Imogen Mitchell,[5] Levon M. Khachigian,[6] Ross W. Stephens,[7] Mark von Itzstein,[2] and Christopher R. Parish[1*]

[1]ACRF Department of Cancer Biology and Therapeutics, The John Curtin School of Medical Research, The Australian National University, Canberra ACT 2601 Australia

[2]Institute for Glycomics, Griffith University, Gold Coast, QLD 4222, Australia

[3]Eccles Institute of Neuroscience, John Curtin School of Medical Research, Australian National University, Canberra, ACT 2601, Australia

[4]Illawarra Health and Medical Research Institute, Wollongong, 2500, Australia

[5]Intensive Care Unit, The Canberra Hospital, Garran, ACT, 2605, Canberra

[6]Vascular Biology and Translational Research, School of Medical Sciences, University of New South Wales, Sydney NSW 2052, Australia

[7]Department of Applied Mathematics, Research School of Physics and Engineering, The Australian National University, Canberra ACT 2601, Australia

[8]These authors contributed equally: Connor H. O'Meara, Lucy A. Coupland, Farzaneh Kordbacheh, Benjamin J. C. Quah and Chih-Wei Chang

[*]e-mail: christopher.parish@anu.edu.au

Table of Contents

Supplementary Figures 1–11 (Biology) 3

Supplementary Chemistry 16

Materials and Methods 16

Synthetic Routes for the Preparation of Sulfated Sugars 17

List of compound structures, numbers and trivial names 17

General procedure for per-*O*-sulfation of free sugars 17

General synthetic scheme for the preparation of methyl per-*O*-sulfonato-β-D-glycosides 18

General procedure A for per-*O*-sulfation (co-solvent approach) 18

General procedure B for per-*O*-sulfation (conventional approach) 18

General procedure C for one-pot per-*O*-acetylation and bromination 19

General procedure D for β-glycosylation using Ag_2CO_3 as promoter 19

General procedure E for Zemplén de-*O*-acetylation 19

Preparation of methyl per-*O*-sulfonato-α-D-cellobioside (S3) 20

Synthetic Routes for the Preparation of a Methyl β-D-cellobioside (mCB) Library 20

General procedure F for 4′,6′-*O*-benzylidenation of mCB 21

General procedure G for benzylation 21

General procedure H for removal of benzylidene acetal 21

General procedure I for hydrogenolysis 21

General procedure J for TBDPS protection 22

General procedure K for removal of TBDPS group 22

Synthetic Procedures, NMR, HPLC and MS Data 23

Glucose per-*O*-sulfate S1 23

Methyl β-D-glucoside per-*O*-sulfate S2 24

Methyl α-D-cellobioside per-*O*-sulfate S3 26

Methyl β-D-cellobioside per-*O*-sulfate (mCBS) S4 27

Lactose per-*O*-sulfate S5 29

Methyl β-D-lactoside per-*O*-sulfate S6 30

Lactulose per-*O*-sulfate S7 32

Xylobiose per-*O*-sulfate S8 33

Methyl β-D-xylobioside per-*O*-sulfate S9 35

Methyl β-D-maltotrioside per-*O*-sulfate S10 36

Melezitose per-*O*-sulfate S11 37

4',6'-di-*O*-sulfated mCB S12 39

4',6,6'-tri-*O*-sulfated mCB S13 40

2,2',3,3'-tetra-*O*-sulfated mCB S14 41

2,2',3,3',6-penta-*O*-sulfated mCB S15 43

References 44

Properties of mCBS 45

Summary of Pharmacology and Toxicology of mCBS 45

Nonclinical Pharmacology 45

Pharmacokinetics and metabolism in animals 46

Toxicology **47**

Anticoagulant Properties of mCBS **50**

Comparison of the anticoagulant activity of mCBS with LMWH and UFH **51**

Chemical Stability of CBS and mCBS **52**

Supplementary Figures 1–11 (Biology)

a

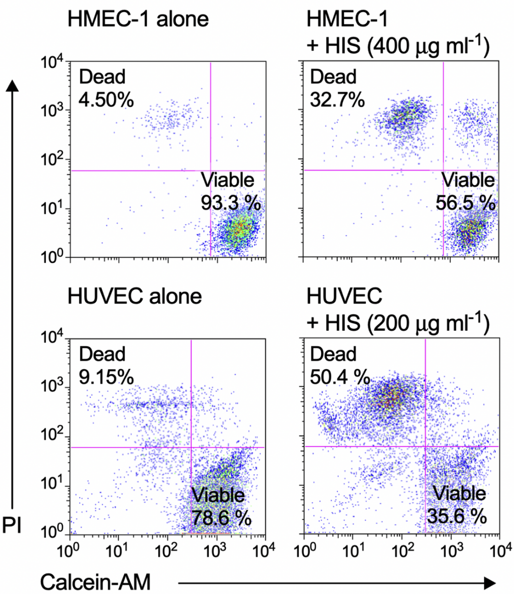

b

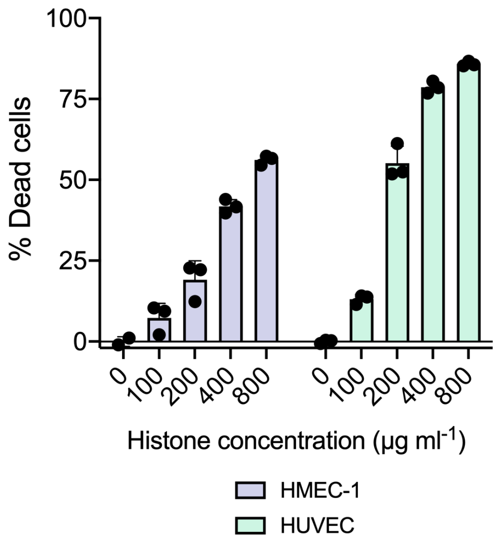

Supplementary Fig. 1 | Flow cytometry-based assay for detecting histone-mediated toxicity for nucleated cells. Suspensions of human microvascular endothelial cells (HMEC-1) or human umbilical vein endothelial cells (HUVEC) were incubated with different concentrations of histones (HIS) for 1 h at 37 °C prior to the addition of calcein-AM (viable cells) or propidium iodide (PI)(dead cells) to assess cell survival by flow cytometry. **a,** Representative flow cytometry plots showing, in the presence of histones, an increase in dead (PI positive/calcein-AM negative) and a decrease in viable (PI negative/calcein-AM positive) HMEC-1 and HUVEC. **b,** Depicts concentration dependent killing of HMEC-1 and HUVEC by histones. Data presented as mean ± s.e.m. (n=2-3). Source data are provided as a Source Data File.

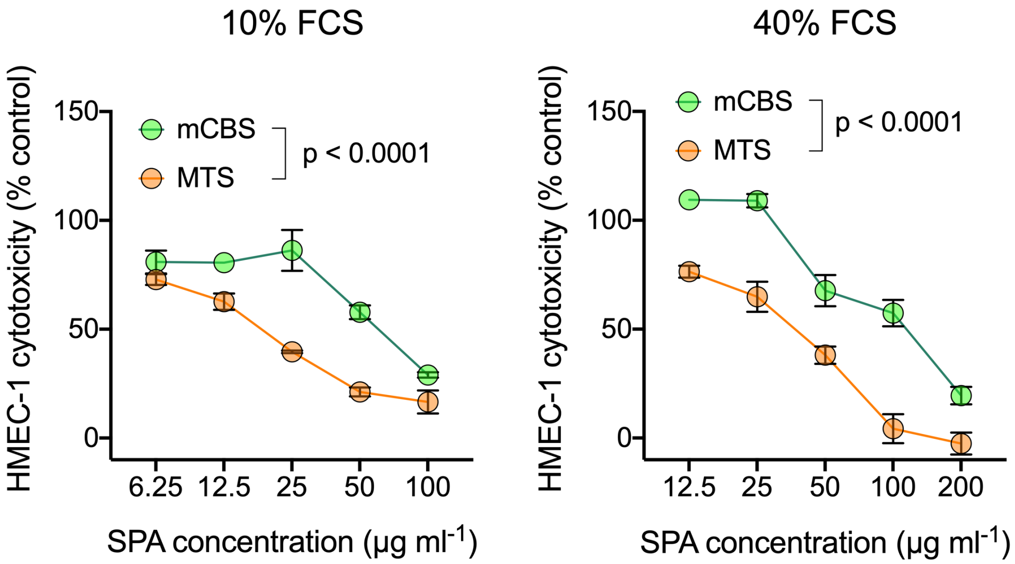

Supplementary Fig. 2 | High serum concentrations do not prevent SPAs from inhibiting histone-mediated cytotoxicity. Suspensions of HMEC-1 were incubated for 1 h at 37 °C with medium containing 10% FCS and 400 µg ml^{-1} of histones (left panel) or 40% FCS and 800 µg ml^{-1} of histones (right panel) and different concentrations of the SPAs mCBS and MTS. Cell survival was then assessed by flow cytometry based on calcein-AM (viable cells) and PI (dead cells) staining. Data presented as mean ± s.e.m (n=3) and analyzed by two-way ANOVA. Source data are provided as a Source Data File.

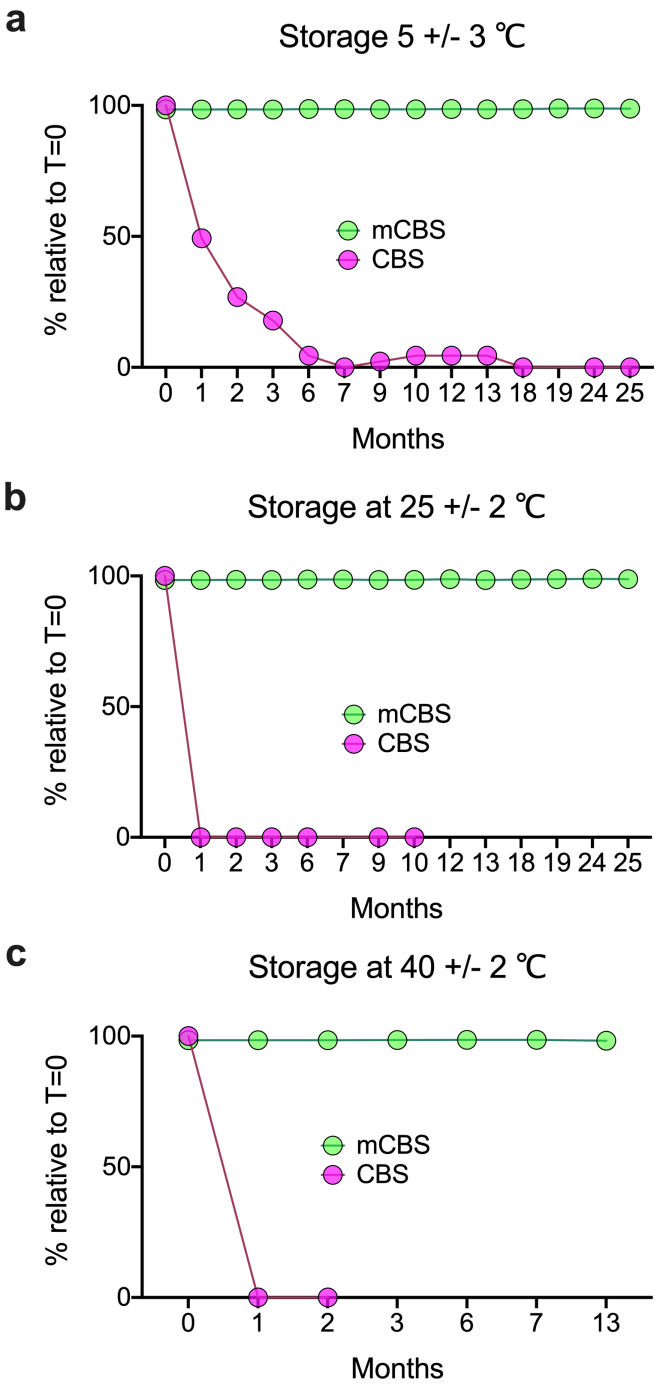

Supplementary Fig. 3 | Differential sensitivity of mCBS and CBS to incubation in aqueous solutions at different temperatures. PBS solutions (pH 7.5) of mCBS and CBS were incubated for up to 25 months at **a,** 5±3 °C, **b,** 25±2 °C or **c,** 40±2 °C and stability of each compound then determined by HPLC. Data expressed as percentage of each compound present at each time point versus the time zero control (T=O). Data provided by Glycosyn.

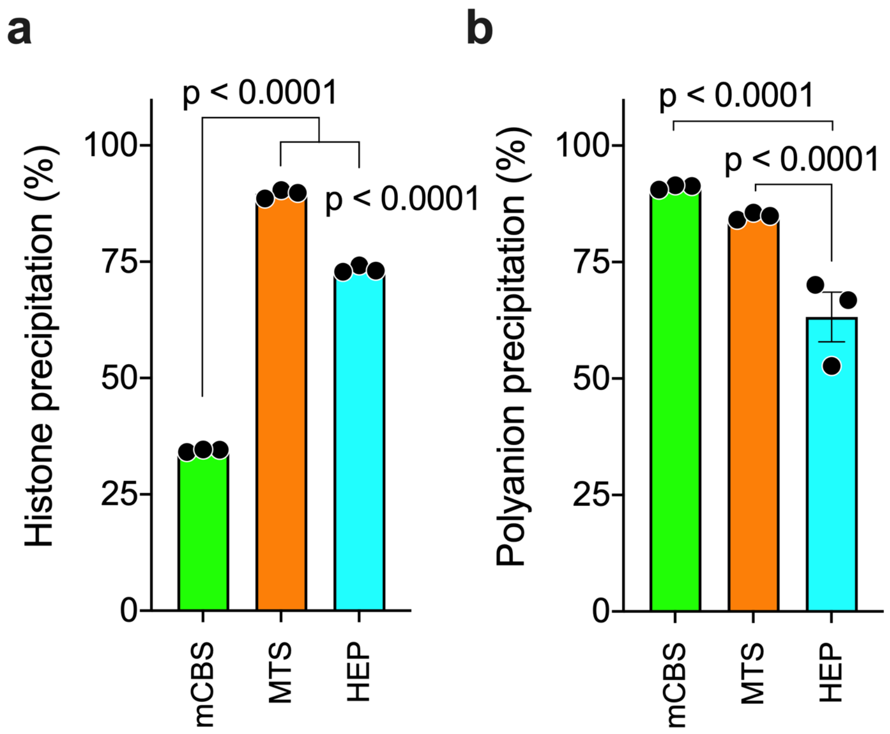

Supplementary Fig. 4 | Precipitation of histones by polyanions. Histones (400 µg $ml^{-1)}$ were mixed with 400 µg ml^{-1} of the polyanions mCBS, MTS and heparin, the resultant precipitates pelleted by centrifugation and the supernatants collected. **a,** Histone concentration in supernatants measured by QUBIT® protein assay and percent histones precipitated calculated. **b,** Polyanion concentration in supernatants measured by the DMMB assay and percent polyanions precipitated calculated. Data presented as mean ± s.e.m. (n=3 biological replicates) and analyzed by two-way ANOVA with Tukey's correction for multiple comparisons. Source data are provided as a Source Data File.

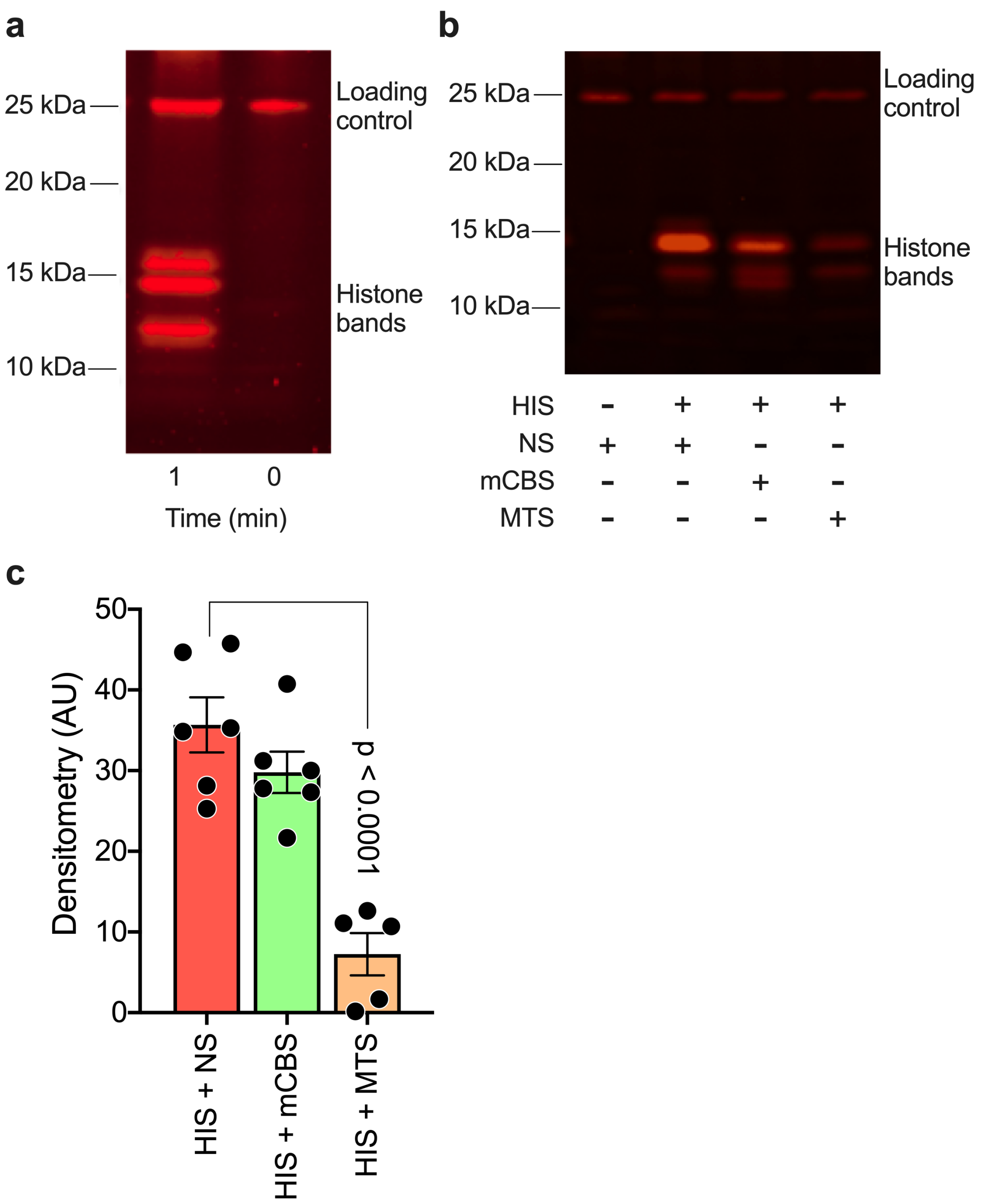

Supplementary Fig. 5 | Detection of free histones in plasma and effect of mCBS and MTS on histone plasma levels. a, Mice injected i.v. with histones (50 mg kg^{-1}) in normal saline (NS) had their blood collected 1 min post injection. Free histones present in the resultant plasma were absorbed to heparin coupled beads, the eluted histones run on SDS-PAGE and visualized with the Sypro Ruby stain. The 0 min sample represents plasma collected from mice injected with NS alone. A 25 kDa unidentified, heparin-binding, protein present in mouse plasma was used as a loading control (data representative of 3 gels). **b,** Effect of injection of mCBS or MTS (100 mg kg^{-1}) 10 min prior to histones on circulating histone levels 1-2 min after histone injection (data representative of 2 gels). **c,** The effect quantified by densitometry**.** Data presented as mean ± s.e.m. (n=5-6 mice/group) and analyzed by one-way ANOVA with Tukey's correction for multiple comparisons. Source data are provided as a Source Data File.

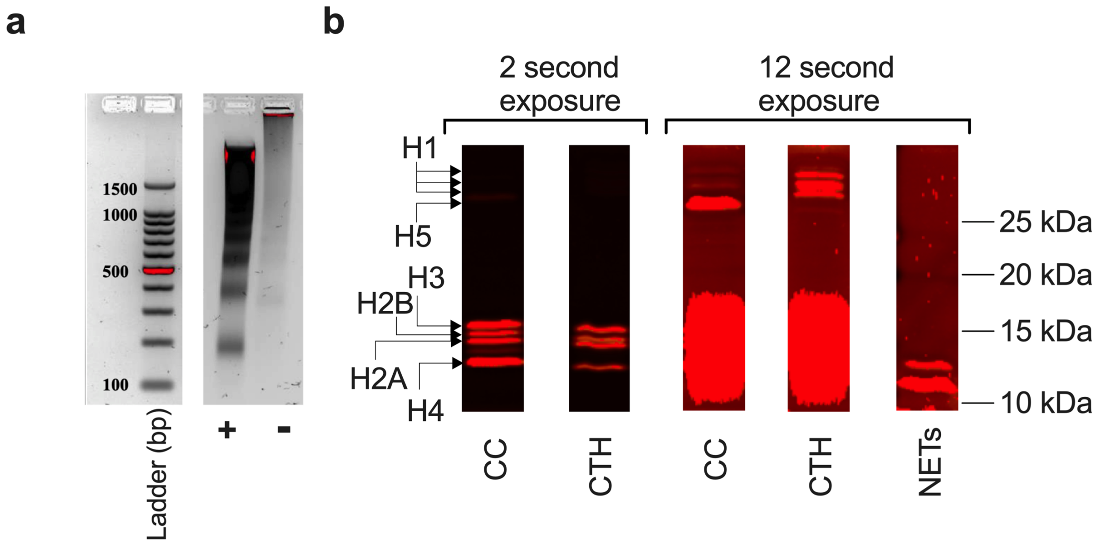

Supplementary Fig. 6 | Composition of different histone preparations. a, Agarose gel electrophoresis of partially micrococcal nuclease digested chicken RBC chromatin before (-) and after (+) proteinase K digestion (data representative of 2 gels). **b,** Histones obtained from different sources were subjected to SDS-PAGE on 4-20% tris-glycine gels and stained with Sypro Ruby protein gel stain. Representative gels, from left to right, are chicken RBC chromatin (CC) and calf thymus histones (CTH) (both 2 seconds exposure), the same CC and CTH samples exposed for 12 seconds to detect presence of CC H5 (H1 analogue in chicken RBC) and three CTH H1 isoforms. Final gel depicts histones detected in a human NET preparation after 12 seconds exposure. Note that with the NET preparation there was a reduction in mol. wt. and number of histone bands detected, indicating that substantial proteolysis of the NET-associated histones has occurred. There was insufficient protein in the NET sample (~24-fold lower content of histones than was detected in the CC sample) to allow detection of human H1 bands (data representative of 10 gels). Source data are provided as a Source Data File.

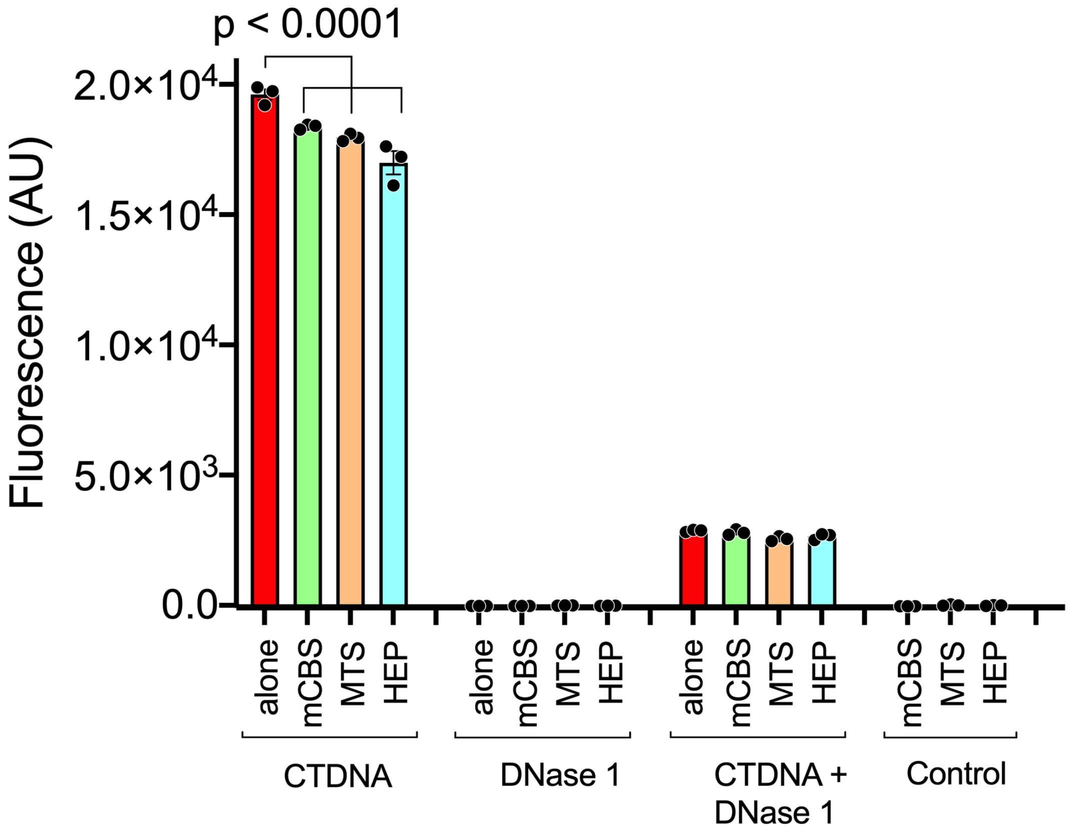

Supplementary Fig. 7 | DNase1 enzymatic activity is not inhibited by heparin or the SPAs mCBS and MTS. Calf thymus DNA (CTDNA) (500 ng ml^{-1}) was incubated for 10 min at 37 °C alone or with DNase1 (1 µg ml^{-1}) and in the presence or absence of heparin or the SPAs mCBS and MTS (100 µg ml^{-1}). DNA content of samples was quantified using PicoGreen, a highly sensitive fluorescent DNA detection reagent. Under the experimental conditions described above ~85% of the CTDNA was degraded and mCBS, MTS or heparin had no significant effect on the amount of undegraded DNA present at the end of the assay, although in the presence of each of the three polyanions there was slight inhibition of uptake of the PicoGreen dye by the undegraded CTDNA control (~5-13%, heparin>MTS>mCBS). Data presented as mean ± s.e.m. (n=3 biological replicates) and analyzed by two-way ANOVA with Tukey's correction for multiple comparisons. Source data are provided as a Source Data File.

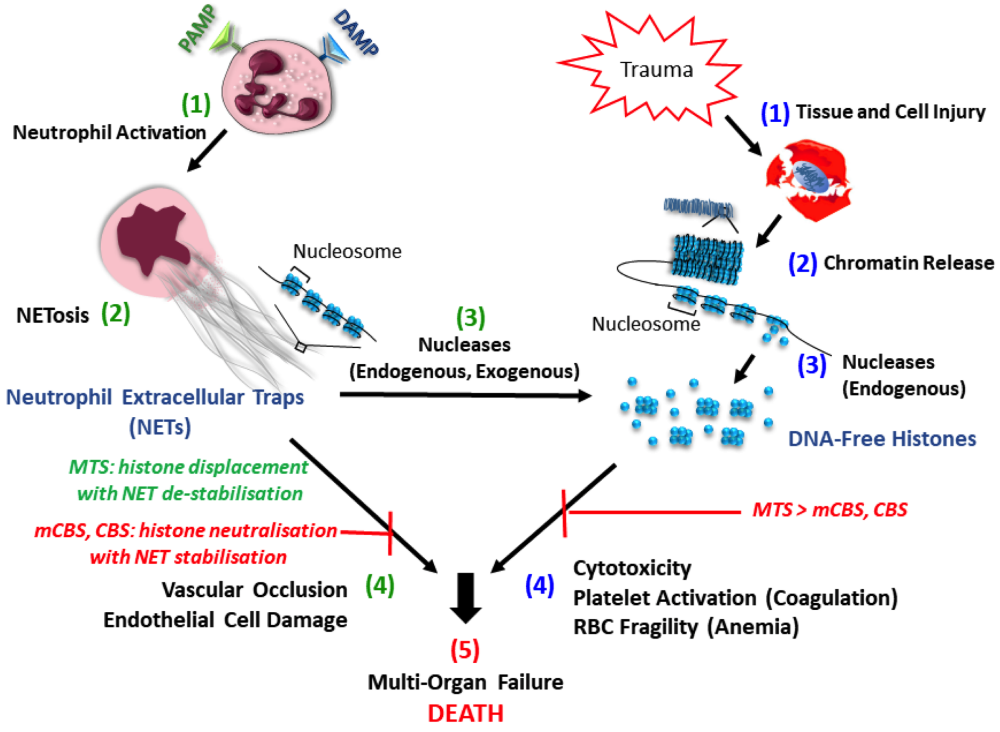

Supplementary Fig. 8 | Effect of different small polyanions (SPAs) on the pathological activity of extracellular histones. Histones, due to their cationic nature, are highly pathological when released extracellularly, either free or DNA-associated. This schematic depicts extracellular histones being derived from two different sources, (1) activated neutrophils (green numbering) and (1) tissue and cell injury (blue numbering). In the case of neutrophils, activation occurs via recognition of pathogen-associated or damage-associated molecular patterns (respectively PAMPs and DAMPs), and (2) results in extrusion (NETosis) of histone-rich chromatin as complex networks called **neutrophil extracellular traps (NETs)**. In contrast, localized or extensive trauma results in a failure to clear damaged cells and the accumulation of histone-rich extracellular chromatin within tissues and the associated microvascular circulation (2). Such a situation (not shown in the diagram) also results in the release of host DAMPs that activate neutrophils to produce NETs. Nucleases, in the case of NETs either endogenous or exogenous (pathogen-derived) (3) or, in the case of tissue and cell injury, endogenously derived (3), degrade histone-associated DNA and release **DNA-free histones** from NETs and trauma-derived chromatin. NETs cause vascular occlusion and endothelial cell (EC) damage (4) with mCBS/CBS reducing these effects by neutralizing histones *in-situ* without causing NET destabilization. In contrast, MTS displaces histones from NETs/chromatin causing their destabilization and increased exposure of NET-associated toxins. Partial digestion of destabilized NETs by endogenous DNases may result in dissemination of NET fragments with downstream microvascular obstruction and tissue damage. Similarly, DNA-free histones are highly pathological but effectively blocked by the SPAs we have developed (4), with both forms of extracellular histones being capable of causing multi-organ failure and death, albeit by different mechanisms (5). Elements of this figure were obtained from the Public Domain source, Servier Medical Art (https://smart.servier.com) and is licensed under a Creative Commons Attribution 3.0 Unported License.

CBS, cellobiose per-*O*-sulfate; ***mCBS***, methyl β-cellobioside per-*O*-sulfate; ***MTS***, maltotriose per-*O*-sulfate.

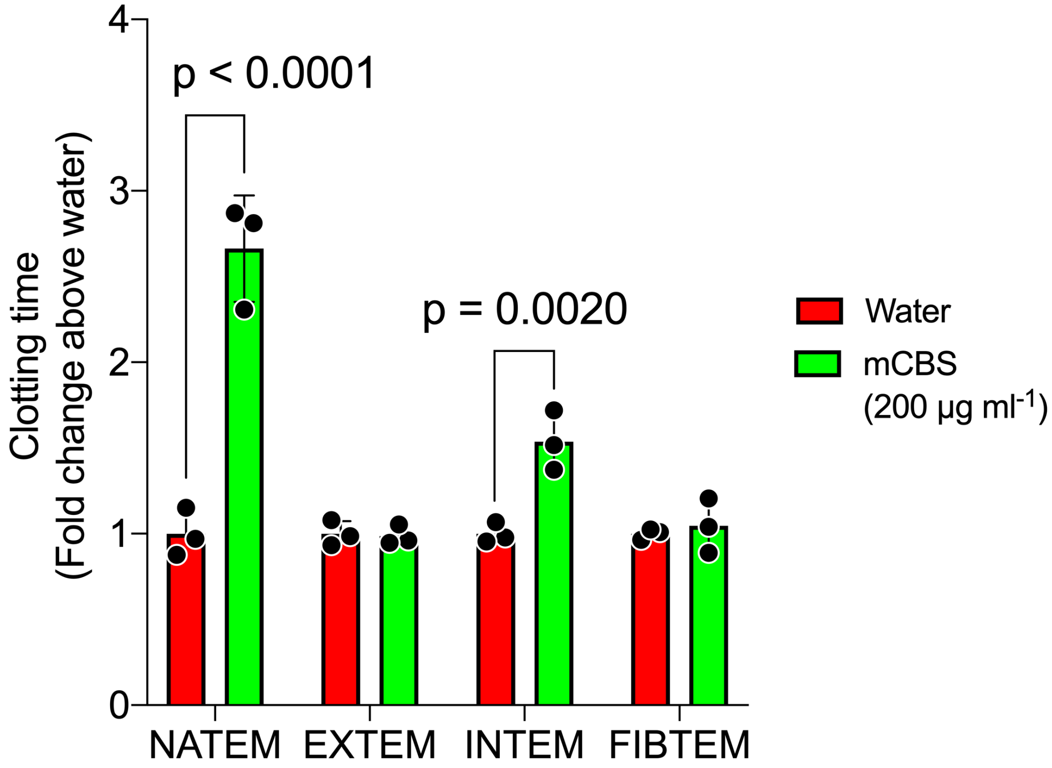

Supplementary Fig. 9 | Effect of mCBS on clotting time. Blood from a healthy unmedicated volunteer was collected into a sodium citrate vacutainer and analysed within 2 h of collection using rotational thromboelastometry (ROTEM). Whole blood (300 µl) was supplemented with mCBS 200 µg ml^{-1} in water or an equivalent volume of water for 1 min then loaded onto the ROTEM machine, according to the manufacturer's instructions, with the NATEM (non-activated), EXTEM (tissue factor activated), INTEM (contact pathway activated) and FIBTEM (tissue factor activated + cytochalasin-D neutralization of platelets) assays performed. Results of each assay (3 replicates) are presented as the fold-change above the value obtained in the water control sample run at the same time. Data presented as mean ± s.e.m. and analyzed by two-way ANOVA with Sidak's correction for multiple comparisons. Source data are provided as a Source Data File.

a

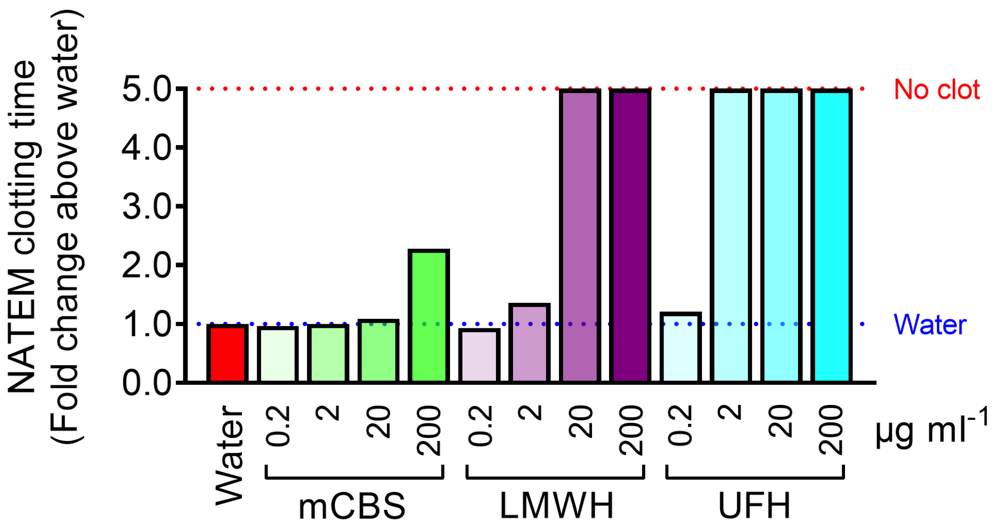

b

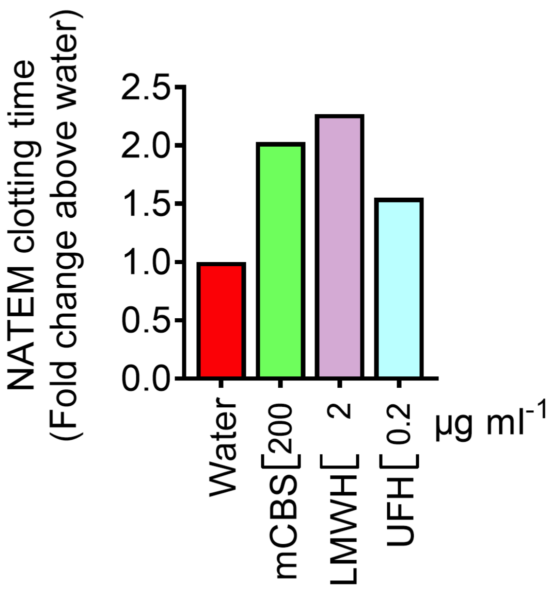

Supplementary Fig. 10 | Comparison of anticoagulant effect of mCBS with low-molecular weight- (LMWH) and unfractionated heparin (UFH). a, Blood from a healthy unmedicated volunteer was collected into a sodium citrate vacutainer and analysed within 2 h of collection using rotational thromboelastometry (ROTEM). Whole blood (300 µl) was supplemented with a titration of compounds in water or an equivalent volume of water for 1 min then loaded onto the ROTEM machine according to the manufacturer's instructions with the NATEM (non-activated) assay used due to its greater sensitivity to heparins (Supplementary Fig 9). **b,** as in **a** however a direct comparison was made between the compounds at the concentrations indicated at the same time rather than across assays. Results are expressed as a percentage of the water control sample run at the same time. Source data are provided as a Source Data File.

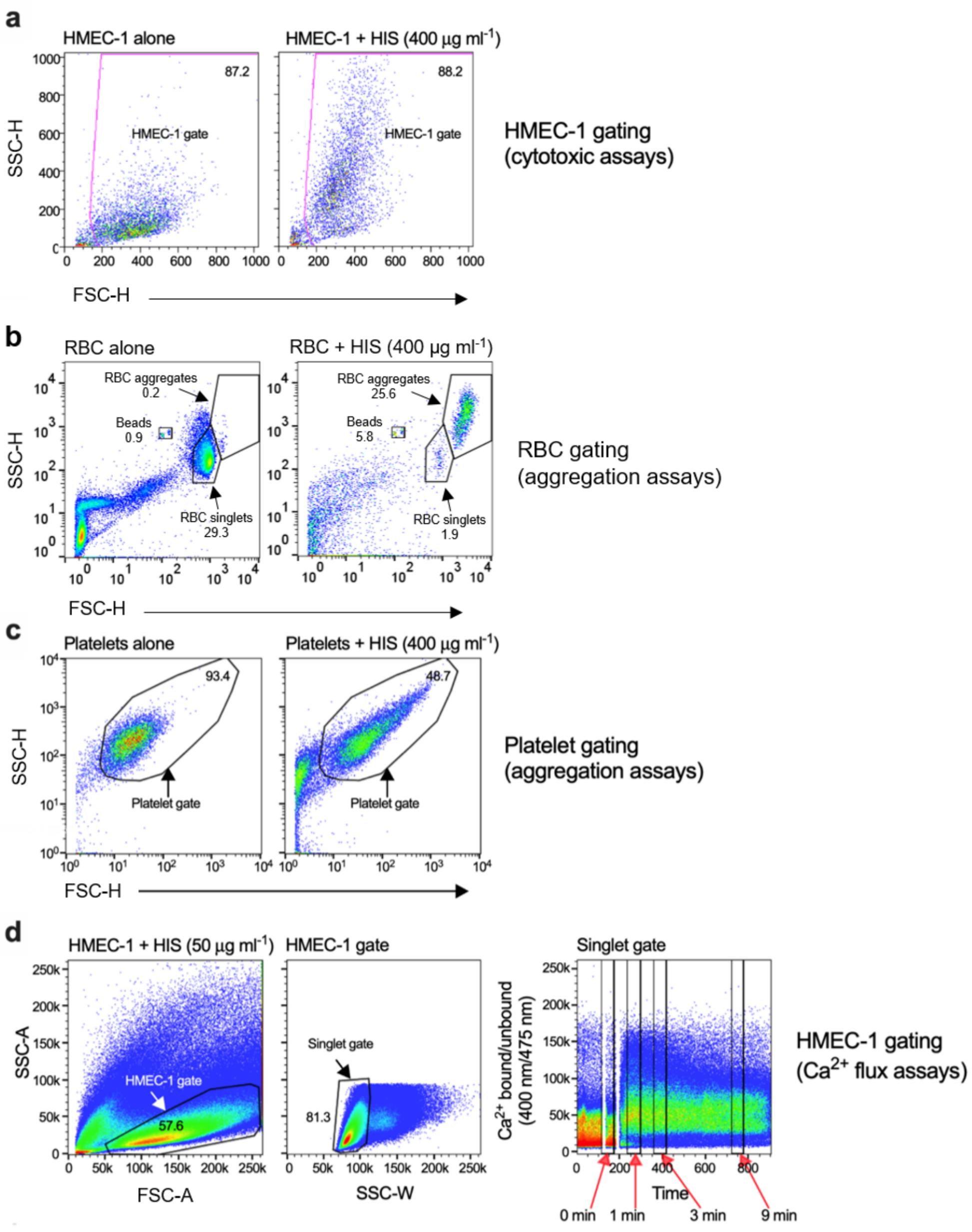

Supplementary Fig. 11 | Initial gating strategies, based on forward (FSC) and side (SSC) scatter, for FACS data. **a,** Gating strategy to remove debris from HMEC-1 cytotoxicity studies (with and without histone treatment) as described in Supplementary Fig. 1, Fig. 1a,c,d and Fig. 3a. **b,** Gating strategy to assess RBC aggregation (with and without histone treatment) as described in Fig. 2a,c,d. **c**, Gating strategy to assess platelet aggregation (with and without histone treatment) as described in Fig. 2e,f. **d,** Gating strategy to assess histone-mediated HMEC-1 Ca^{2+} flux in viable cells (with and without CBS/MTS treatment) as described in Fig. 3d. After FSC/SSC gating for viable HMEC-1, singlets gated and Ca^{2+} flux measured for 1 min after addition of CBS or MTS (0 min), and 1 min, 3 min and 9 min after histone addition.

Supplementary Chemistry

Materials and Methods

Unless stated otherwise, reactions were performed in oven-dried glassware with reagents and dry solvents purchased from commercial sources. Thin-layer chromatography (TLC) was performed using aluminium plates coated with silica gel 60 F254 (Merck). Reactions monitored by TLC were visualized by UV light and heated upon reaction with ethanolic H_2SO_4 (5% v/v). Flash chromatography was performed using Silica Gel 60 (0.040–0.063 mm).

NMR spectra were recorded on a Bruker Avance 400 or 600 MHz (Ultra-shield) spectrometer. NMR data acquisition and processing were performed with Mestrenova software. ^{1}H NMR and ^{13}C NMR chemical shifts (δ) are reported in parts per million, relative to the residual solvent peak as an internal reference. [$CDCl_3$: 7.26 (s) for ^{1}H; 77.16 (t) for ^{13}C; CD_3CN: 1.94 (pent) for ^{1}H; 1.4, 118.7 for ^{13}C; CD_3OD: 3.31 (pent) for ^{1}H; 49.0 (sept) for ^{13}C; D_2O: 4.79 (s) for ^{1}H]. 2D COSY and HSQC experiments were accessed to support assignments.

IR spectra were recorded on a Bruker ALPHA-P FT-IR spectrometer. Low resolution mass spectra (LRMS) were acquired in either positive or negative ion mode as indicated on a Bruker Daltonics Esquire 3000 ESI MS; high-resolution mass spectra (HRMS) data were acquired at either Griffith University's FTMS Facility on a Bruker QTOF mass spectrometer or at the Smart Water Research Centre on an Agilent 6530 Q-TOF mass spectrometer using Agilent Jetstream ESI.
All final compounds were purified by HPLC using a gradient of H_2O/CH_3CN in 10 mM ammonium acetate at 1 mL/min through a Phenomenex 5 µm C18 guard column.

A Luna 3 µm hydrophilic liquid interaction chromatography (HILIC) column (4.6 × 150 mm^2, 200 Å, Phenomenex, Column ID no.: A-HILIC-MVI#5) was used to analyze the product purity as detected by ELSD. HPLC analysis was conducted using a linear gradient of **A:** 5 mM ammonium formate in water and **B:** 5 mM ammonium formate in 95% acetonitrile + 5% water at a flow rate of 1.0 mL/min.

Synthetic Routes for the Preparation of Sulfated Sugars

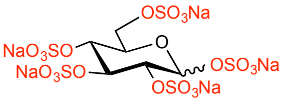

S1 (Glucose per-*O*-sulfate)

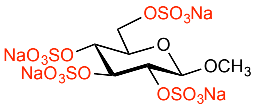

S2 (Methyl β-D-glucoside per-*O*-sulfate)

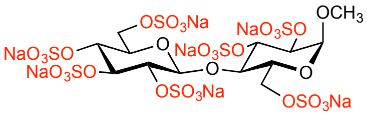

S3 (Methyl α-D-cellobioside per-*O*-sulfate)

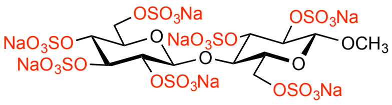

S4 (Methyl β-D-cellobioside per-*O*-sulfate, 2,2',3,3',4',6,6'-hepta-*O*-sulfated, mCBS)

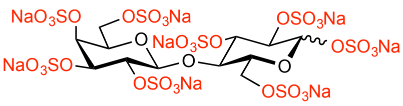

S5 (Lactose per-*O*-sulfate)

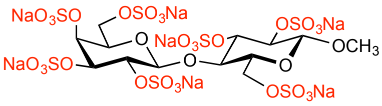

S6 (Methyl β-D-lactoside per-*O*-sulfate)

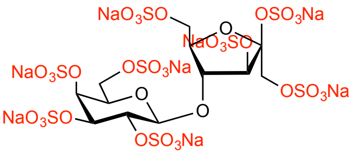

S7 (Lactulose per-*O*-sulfate)

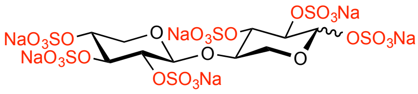

S8 (Xylobiose per-*O*-sulfate)

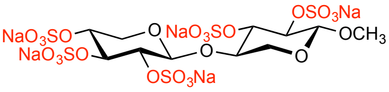

S9 (Methyl β-D-xylobioside per-*O*-sulfate)

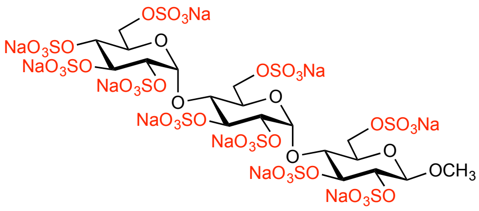

S10 (Methyl β-D-maltotrioside per-*O*-sulfate)

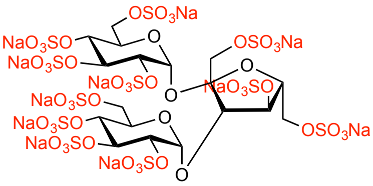

S11 (Melezitose per-*O*-sulfate)

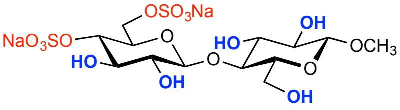

S12 (4',6'-di-*O*-sulfated mCB)

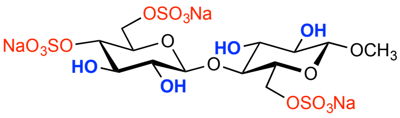

S13 (4',6,6'-tri-*O*-sulfated mCB)

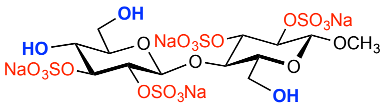

S14 (2,2',3,3'-tetra-*O*-sulfated mCB)

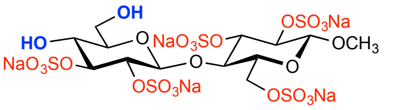

S15 (2,2',3,3',6-penta-*O*-sulfated mCB)

Supplementary Fig. 12 | List of compound structures, numbers and trivial names.

General procedure for per-*O*-sulfation of free sugars

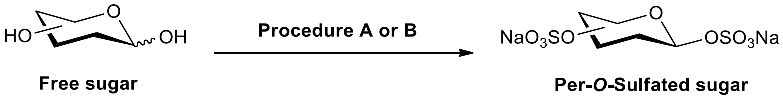

Supplementary Scheme 1 | Synthesis of per-*O*-sulfated sugars.

General synthetic scheme for the preparation of methyl per-*O*-sulfonato-β-D-glycosides

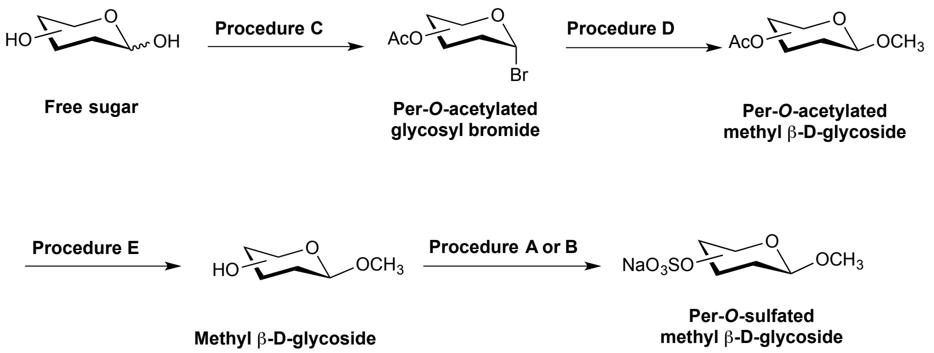

Supplementary Scheme 2 | Synthesis of per-*O*-sulfated glycosides.

General procedure A for per-*O*-sulfation (co-solvent approach)

Standard Protocol: To the polyol sugar (1 mmol) was added sulfur trioxide-trimethylamine complex (SO_3•TMA, 1.5 to 5 equiv per OH), followed by the addition of anhydrous DMF (1.2 mL/per 1 mmol of SO_3•TMA) and anhydrous DCE. The preferred reaction concentration is between 10 to 50 mM. The resulting suspension was heated under Argon at 80 - 90 ^{o}C for 10 min to 1 h. Upon cooling, the mixture was co-evaporated with chlorobenzene to remove DMF under reduced pressure. The crude residue was then dissolved in water and filtered through a pad of cotton. The transparent and brownish liquid was further purified through size exclusion chromatography (SEC) on Sephadex G-25 using water as the eluent. Combined fractions were concentrated to obtain the final sulfated compound which was dissolved in de-ionized water and directly subjected to ion-exchange chromatography (DOWEX 50Wx8, Na^+ form). The combined fractions were lyophilized to yield the final sulfated product as a sodium salt.

General procedure B for per-*O*-sulfation (conventional approach)

Standard Protocol: To a solution of the starting material in anhydrous DMF (60 mL/per mmol of starting material) was added sulfur trioxide–trimethylamine complex (SO_3•TMA, 1.5 to 5 equiv per OH) under Argon. The mixture was stirred at 50 - 60 °C until reaction completion was indicated by ^{1}H-NMR spectroscopy. Upon cooling, the reaction mixture was quenched with Et_3N and co-evaporated with chlorobenzene to remove DMF (bath temperature is not over 30 °C) under reduced pressure. The crude residue was dissolved in water and filtered through a pad of cotton. The transparent and brownish liquid was further purified through size exclusion chromatography (SEC)

on Sephadex G-25 using water as eluent. Combined fractions were concentrated to obtain the final sulfated compound as a triethylammonium salt which was dissolved in de-ionized water and directly subjected to ion-exchange column (DOWEX 50Wx8, Na^+ form). The combined fractions were lyophilized to yield the final sulfated product as a sodium salt.

General procedure C for one-pot per-*O*-acetylation and bromination

To the mixture of free sugar (20 mmol) and glacial acetic acid (100 mL) was added acetyl bromide (1.5 equiv per OH) at room temperature. The resultant creamy mixture was subsequently heated at 60 °C for 45 - 55 min until the reaction mixture turned clear, which indicated that the reaction was complete. The hot reaction mixture was carefully poured into a beaker pre-charged with cracked ice (300 g). The mixture was stirred until a white solid precipitated (~10 min), upon which another portion of cold water (60 mL) was added and the mixture stirred for a further 10 min. The suspension was then filtered through a sintered funnel and washed with cold water (30 mL x 3) to obtain the precipitated product that was then further dissolved in DCM (100 mL). The DCM layer was washed with brine, dried over Na_2SO_4, filtered and concentrated under reduced pressure at below 35 °C to yield the target glycosyl bromide that was then directly used in the following glycosylation reaction.

General procedure D for β-glycosylation using Ag_2CO_3 as promoter

To a mixture of per-*O*-acetylated glycosyl bromide (25 mmol), anhydrous DCM (80 mL), anhydrous MeOH (80 mL), and activated 3 Å molecular sieves (7 g) was added silver carbonate (Ag_2CO_3, 7.5 g, 27.5 mmol, 1.1 equiv). The resultant mixture was stirred in the absence of light for 16 h. The reaction mixture was then purified through a plug of silica and eluted with EtOAc. The collected fractions were concentrated to give the crude product as a brownish solid that was directly used in the next step.

General procedure E for Zemplén de-*O*-acetylation

To the suspension mixture of per-*O*-acetylated sugar in anhydrous MeOH (100 mL) was added a small piece of Na (172 mg, 7.5 mmol, 0.3 equiv) at room temperature. The mixture was then stirred overnight in order to ensure completion of de-*O*-acetylation. The final solution was concentrated and dried under vacuum overnight to obtain the crude methyl glycoside that was then directly used for sulfation.

Preparation of methyl per-*O*-sulfonato-α-D-cellobioside (S3)

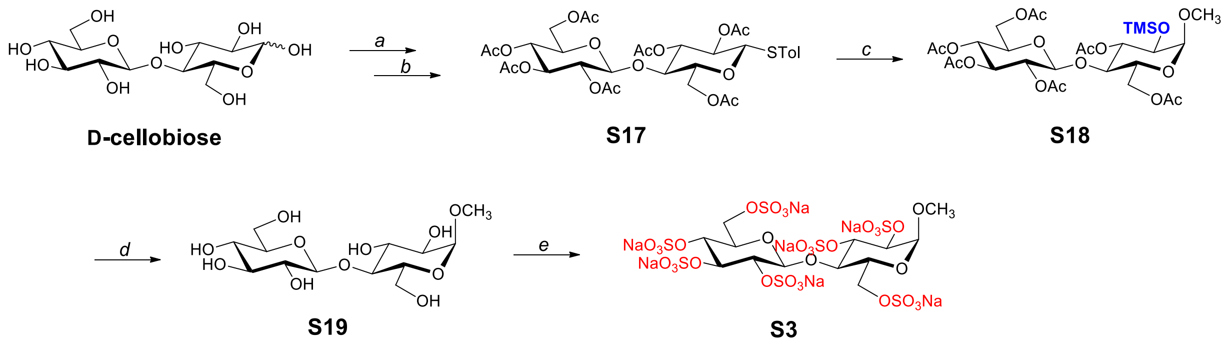

Supplementary Scheme 3 | Reagent and reaction conditions. (a) Ac_2O, ZnI_2, 80 °C, 87%. (b) TMSSTol, DCE, 70 °C, 61% over 2 steps. (c) MeOH, NIS/TMSTOf, DCM, 0 °C, 25%. (d) NaOMe, MeOH, 99%. (e) SO_3•TMA, DMF/DCE (2/1, v/v), 90 °C, 18%.

Synthetic Routes for the Preparation of a Methyl β-D-cellobioside (mCB) Library

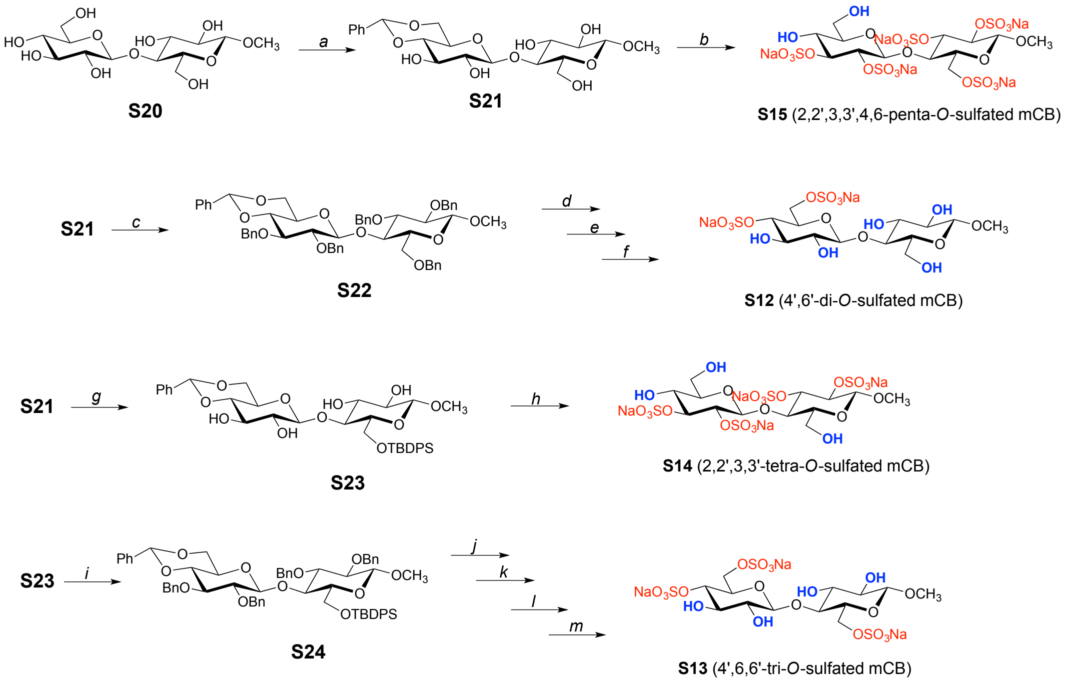

Supplementary Scheme 4 | Reagent and reaction conditions. (a) Benzaldehyde dimethyl acetal, CSA, DMF, 50 °C, 87%. (b) SO_3•TMA, DMF/DCE (3/1, v/v), 70 °C, 4%. (c) BnBr, NaH, DMF, 0 °C, 60%. (d) TFA, DCM, 0 °C, 56%. (e) SO_3•TMA, DMF/DCE (1/20, v/v), 100 °C. (f) $Pd(OH)_2/C$, H_2, phosphate buffer pH = 7, MeOH, 50 % over two steps. (g) TBDPSCl, Imidazole, Et_3N, DMF, rt, 29%. (h) SO_3•TMA, DMF/DCE (2/3, v/v), 70 °C, 7%. (i) BnBr, NaH, DMF, 0 °C. 60%. (j) TFA, DCM, 0 °C, 56%. (k) AcOH (20 equiv.), 1M TBAF, THF, 50 °C, 56% over two steps (l) SO_3•TMA, DMF/DCE (1/10, v/v), 100 °C. (m) $Pd(OH)_2/C$, H_2, phosphate buffer pH = 7, MeOH, 44 % over two steps.

General procedure F for 4´,6´-*O*-benzylidenation of methyl cellobioside

A solution of substrate, benzaldehyde dimethyl acetal (2.0 equiv), CSA (0.1 equiv) in anhydrous DMF (3 mL for 1 mmol of substrate) was heated at 50 °C under reduced pressure (25 mm Hg) for a period of time until >95% conversion was observed as determined by TLC. The reaction was quenched with triethylamine (TEA, 5 equiv) and DMF removed by rotary evaporation. The resultant crude mixture was diluted with EtOAc, washed with 1% aqueous HCl solution, water, saturated $NaHCO_3$ aqueous solution and brine, dried over Na_2SO_4, filtered and concentrated to obtain the crude product. Further purification was performed by either flash column chromatography or trituration.

General procedure G for benzylation

To a solution of substrate and benzyl bromide (1.1 equiv/per OH) in anhydrous *N*,*N*-dimethylformamide (DMF, 1 mL for 1 mmol of substrate) in an ice bath was portion-wise added 60% NaH (1.2 equiv/per OH). The reaction was allowed to warm up to room temperature until H_2 evolution ceased. The resultant mixture was stirred for a further two hours and the reaction carefully quenched with water at 0 °C. Upon removal of DMF by rotary evaporation, the crude mixture was diluted with EtOAc, washed with 1% aqueous HCl solution, water, saturated $NaHCO_3$ aqueous solution, brine, dried over Na_2SO_4, filtered and concentrated to obtain the crude benzylated product. Further purification was performed by flash column chromatography.

General procedure H for removal of benzylidene acetal

To a solution of benzylidenated substrate (0.3 mmol) in DCM (5 mL) was added trifluoroacetic acid (TFA, 5 equiv) at 0 °C. The solution was gradually warmed to room temperature and stirred for 1-2 h. Upon completion of reaction, TFA was removed by co-evaporation with toluene to furnish the crude product that was then washed with saturated $NaHCO_3$ aqueous solution, brine, dried over Na_2SO_4, filtered and concentrated to obtain the crude 4',6'-diol. Further purification was performed by flash column chromatography to yield the debenzylidenated product.

General procedure I for hydrogenolysis

A solution of protected saccharide and palladium hydroxide on carbon (20 wt. % loading, 10 times the weight of starting material) in phosphate buffer (20 mM, pH = 7.0, 200 µL/per mg of starting material) and MeOH (200 µL/per mg of starting material) was charged with a hydrogen balloon. The resultant mixture was stirred at room temperature for 2 days. The reaction mixture was filtered through celite, and the filtrate was concentrated and purified using a Sephadex G-25 column eluted with water. After concentration, the residue was dissolved in water and passed through an ion-

exchange column of DOWEX 50W-X8 (Na^+ form). The collected fractions were lyophilized to give the target product as a sodium salt.

<u>**General procedure J** for TBDPS protection</u>

To a continuously stirred solution of benzylidenated starting material (0.5 mmol) in DMF (30 mL) at room temperature was added Et_3N (1.5 equiv), imidazole (0.5 equiv) and finally TBDPSCl (1.2 equiv). The mixture was stirred for a further 3 h. Another portion of TBDPSCl (0.6 equiv) was added and the reaction was stirred for another 24 h. The resultant mixture was quenched with MeOH, diluted with EtOAc (200 mL), washed with 1% HCl aqueous solution, water, saturated $NaHCO_3$ aqueous solution, brine, dried over Na_2SO_4, filtered and concentrated to furnish the silylated crude mixture that was further purified using silica column chromatography to yield the pure protected product.

<u>**General procedure K** for removal of TBDPS group</u>

To a solution of protected saccharide (0.31 mmol) in THF (10 mL) was added acetic acid (20 equiv) and tetrabutylammonium fluoride (TBAF, 1.0 M solution in THF, 12 equiv) at room temperature under Ar. The resultant solution was heated at 50 °C overnight. THF and acetic acid were removed under reduced pressure and directly subjected to flash column chromatography on silica gel to afford the desilylated product.

Synthetic Procedures, NMR, HPLC and MS Data

1,2,3,4,6-Penta-*O*-sulfonato-α,β-D-glucopyranose pentasodium salt (S1)

Compound **S1** was prepared from D-glucose in 35% yield following **Procedure B**. **^{1}H NMR** (400 MHz, D_2O) δ 6.04 (d, *J* = 3.5 Hz, 1H, H-1α), 4.70 (dd, *J* = 9.9, 8.9 Hz, 1H, H-3), 4.58 – 4.41 (m, 3H, H-2, H-4, H-6a), 4.34 – 4.23 (m, 2H, H-6b, H-5); **^{13}C NMR** (101 MHz, D_2O) δ 94.77 (C-1), 75.17 (C-3), 73.83 (C-2), 73.68 (C-4), 70.09 (C-5), 66.47 (C-6); **HRMS** (ESI) calculated for $C_6H_{12}O_{21}S_5$ $[M-5H+4Na]^-$ m/z -666.7674, found -666.7672. These data are consistent with those previously reported in literature [1].

Supplementary Fig. 13 | ^{1}H-NMR spectrum of S1 (400 MHz, D_2O).

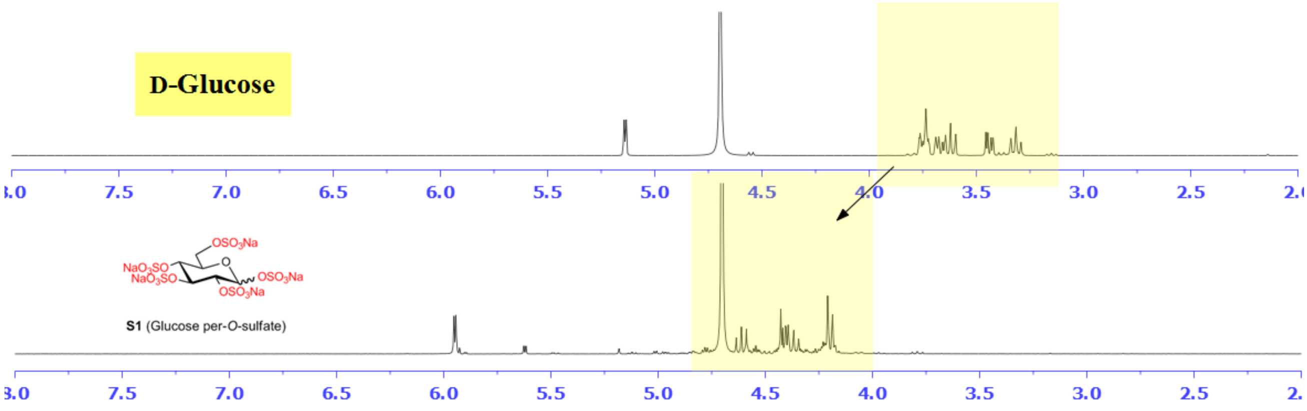

Supplementary Fig. 14 | ^{13}C-NMR spectrum of S1 (100 MHz, D_2O).

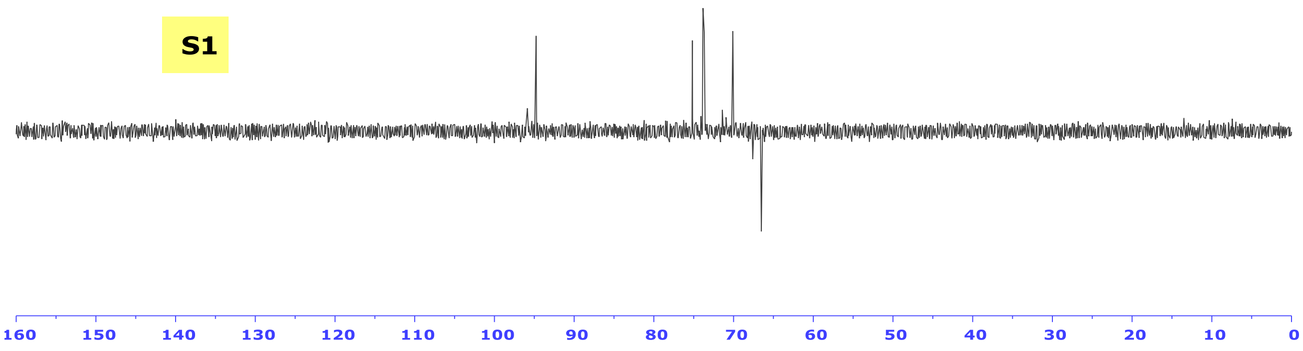

Supplementary Fig. 15 | 2D ^{1}H-^{1}H COSY and 2D ^{1}H-^{13}C HSQC spectrum of S1.

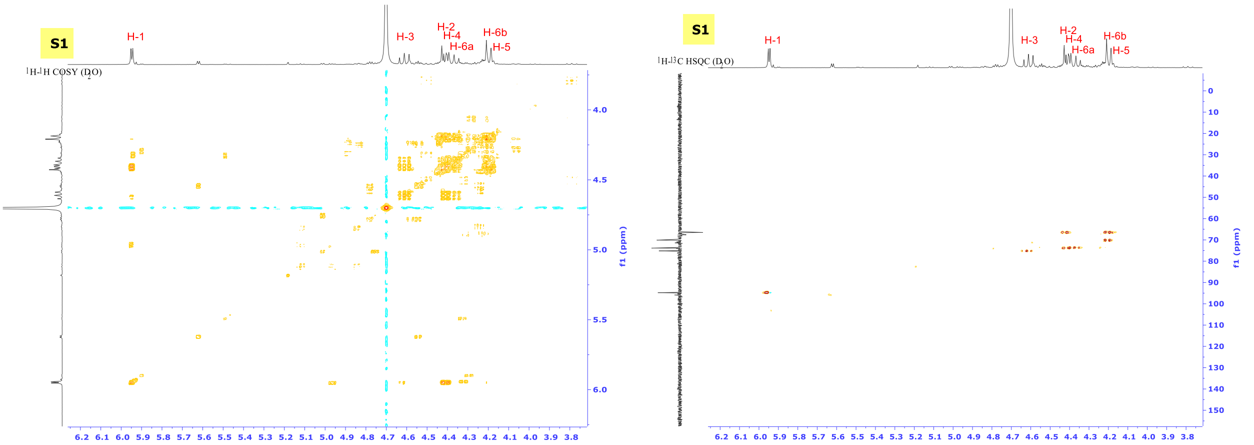

Supplementary Fig. 16 | HPLC analysis and HRMS spectrum of S1.

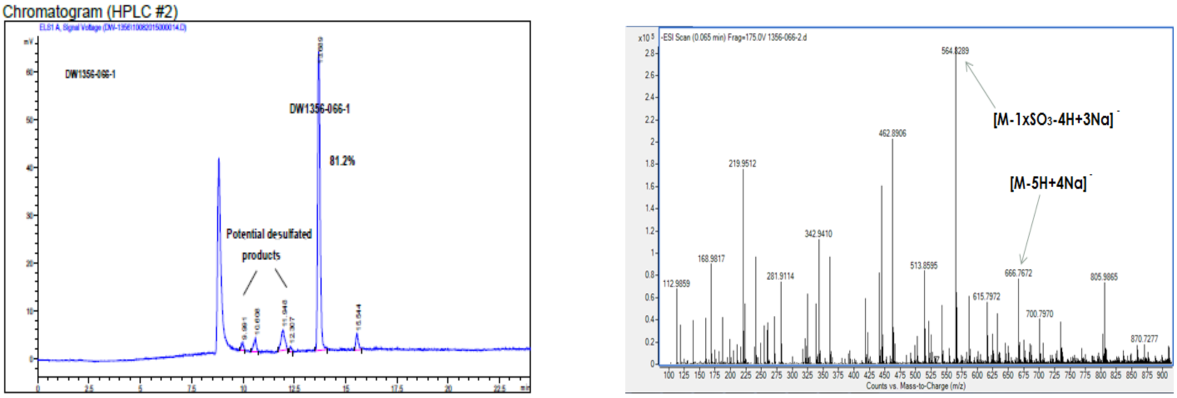

Methyl 2,3,4,6-tetra-*O*-sulfonato-β-D-glucopyranoside tetrasodium salt (S2)

Compound **S2** was prepared from D-glucose in 77% yield following **Procedure C**, **D**, **E** and **A**. **^{1}H NMR** (400 MHz, D_2O) δ 4.83 (d, *J* = 5.1 Hz, 1H, H-1), 4.81 – 4.77 (m, 1H, H-3), 4.56 (appt, *J* = 6.0 Hz, 1H, H-4), 4.48 (dd, *J* = 10.8, 8.1 Hz, 1H, H-6a), 4.46 (appt, *J* = 5.2 Hz, 1H, H-2), 4.28 (dd, *J* = 10.8, 8.1 Hz, 1H, H-6b), , 4.25 – 4.16 (m, 1H, H-5), 3.60 (s, 3H); **^{13}C NMR** (100 MHz, D_2O) δ 101.15 (C-1), 75.96 (C-2), 75.55 (C-3), 73.36 (C-5), 72.52 (C-4), 67.91 (C-6), 57.11 (OCH_3); NMR spectroscopic data are in agreement with published values[2]; **HRMS** (ESI) calculated for $C_7H_{14}O_{18}S_4$ $[M-4H+3Na]^-$ m/z -578.8443, found -578.8456.

Supplementary Fig. 17 | ^{1}H-NMR spectrum of S2 (400 MHz, D_2O).

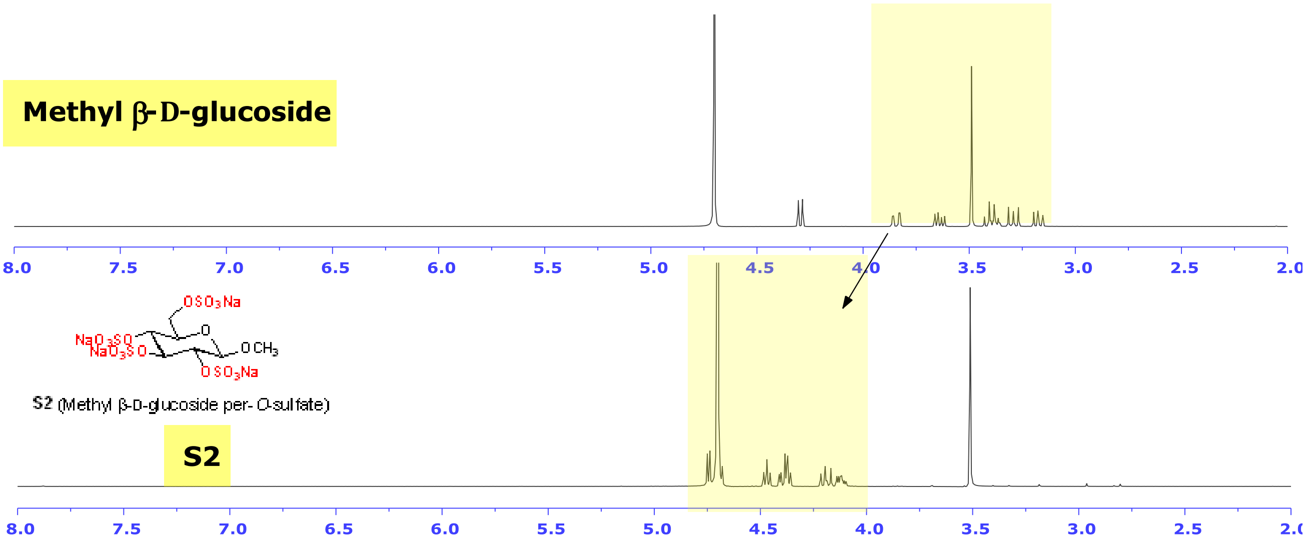

Supplementary Fig. 18 | ^{13}C-NMR spectrum of S2 (100 MHz, D_2O).

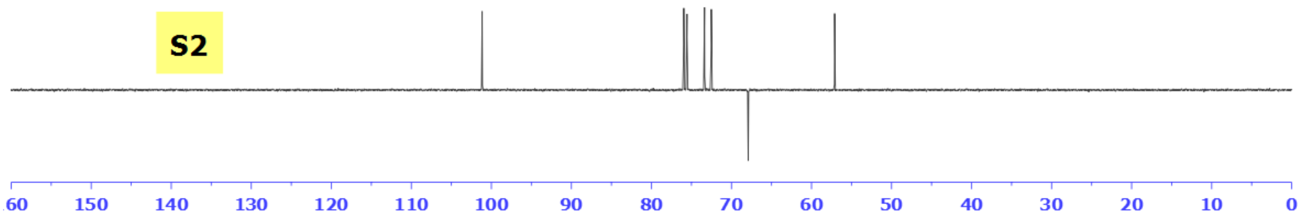

Supplementary Fig. 19 | 2D ^{1}H-^{1}H COSY and 2D ^{1}H-^{13}C HSQC spectrum of S2.

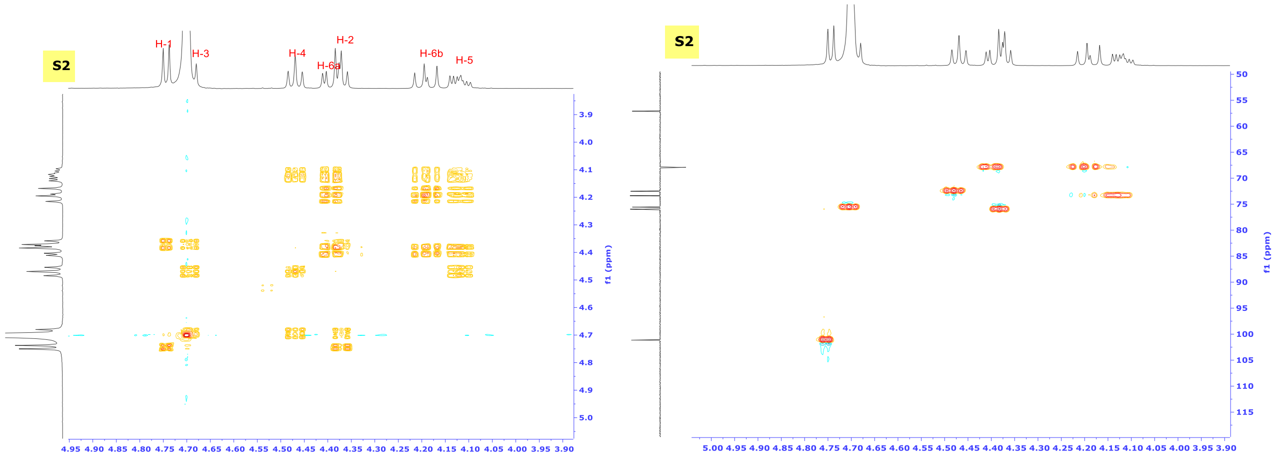

Supplementary Fig. 20 | HPLC analysis and HRMS spectrum of S2.

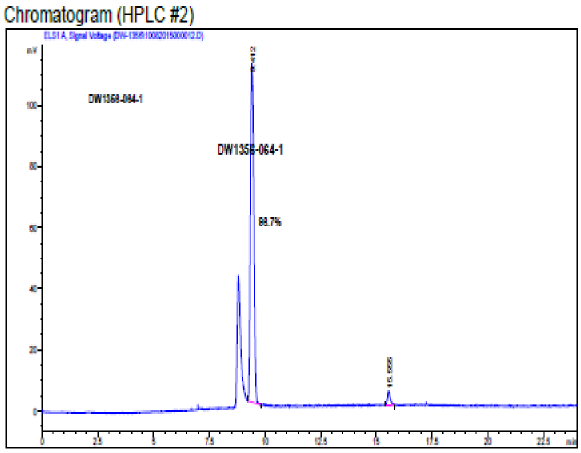

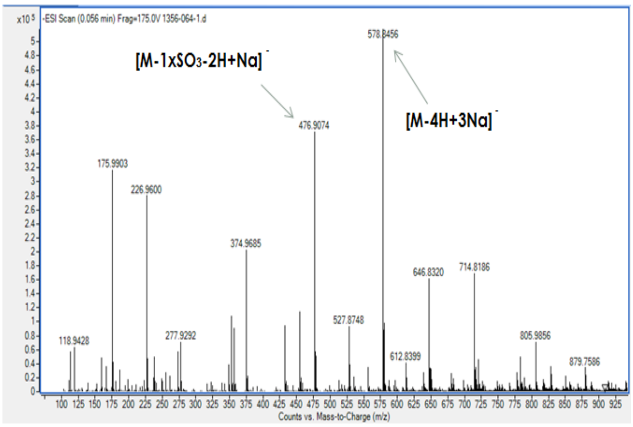

Methyl 2,3,4,6-tetra-*O*-sulfonato-β-D-glucopyranosyl-(1→4)-2,3,6-tri-*O*-sulfonato-α-D-glucopyranoside heptasodium salt (S3)

Compound **S3** was prepared from D-cellobiose in 18% yield following procedures described in **Supplementary Scheme 3**. **^{1}H NMR** (600 MHz, D_2O) δ 5.18 (d, J = 3.6 Hz, 1H, H-1), 4.85 (d, J = 7.4 Hz, 1H, H-1'), 4.67 – 4.62 (m, 2H, H-3, H-6'a), 4.60 (appt, J = 8.1 Hz, 1H, H-3'), 4.56 (dd, J = 11.1, 2.3 Hz, 1H, H-6a), 4.44 – 4.38 (m, 2H, H-4', H-2), 4.37 – 4.31 (m, 2H, H-6b, H-2'), 4.19 (dd, J = 11.3, 7.7 Hz, 1H, H-6'b), 4.08 – 4.03 (m, 2H, H-5, H-4), 4.00 – 3.95 (m, 1H, H-5'), 3.48 (s, 3H, OCH_3); **^{13}C NMR** (150 MHz, D_2O) δ 99.69 (C-1'), 97.09 (C-1), 77.77 (C-2'), 77.74 (C-3'), 76.04 (C-3), 75.24 (C-2), 74.59 (C-4), 73.80 (C-4'), 73.46 (C-5'), 68.52 (C-5), 68.01 (C-6'), 65.90 (C-6), 55.24 (OCH_3); **HRMS** (ESI) calculated for $C_{13}H_{24}O_{32}S_7$ [M-7H+5Na]$^{-2}$ m/z -511.8618, found -511.8611.

Supplementary Fig. 21 | ^{1}H-NMR spectrum of S3 (600 MHz, D_2O).

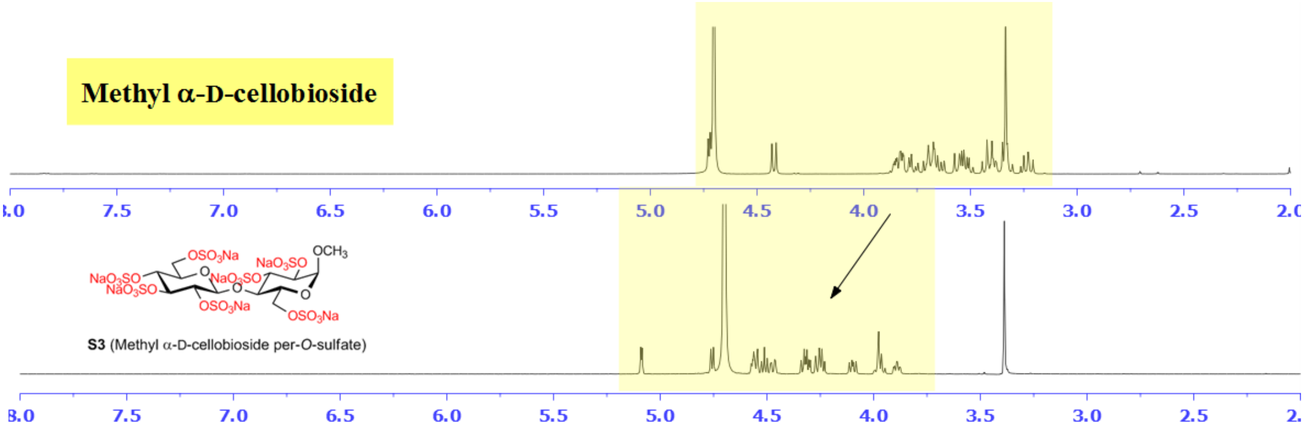

Supplementary Fig. 22 | ^{13}C-NMR spectrum of S3 (150 MHz, D_2O).

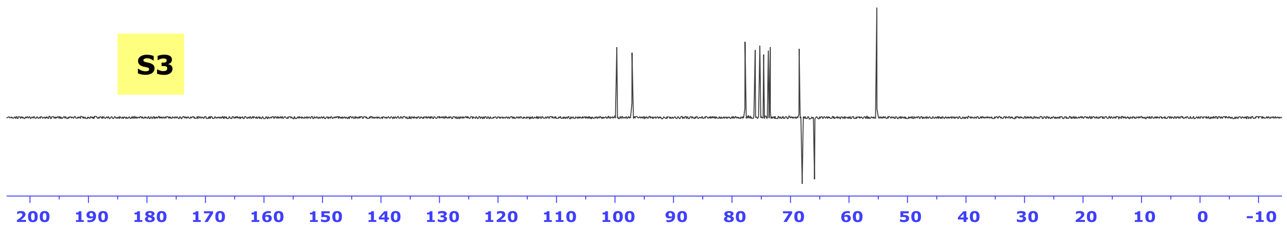

Supplementary Fig. 23 | 2D ^{1}H-^{1}H COSY and 2D ^{1}H-^{13}C HSQC spectrum of S3.

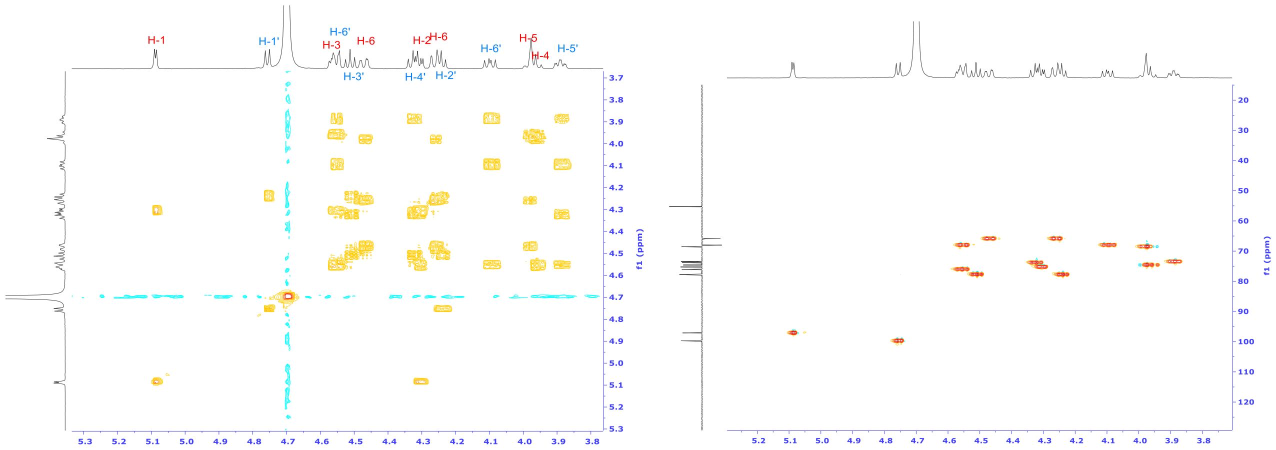

Supplementary Fig. 24 | HPLC analysis and HRMS spectrum of S3.

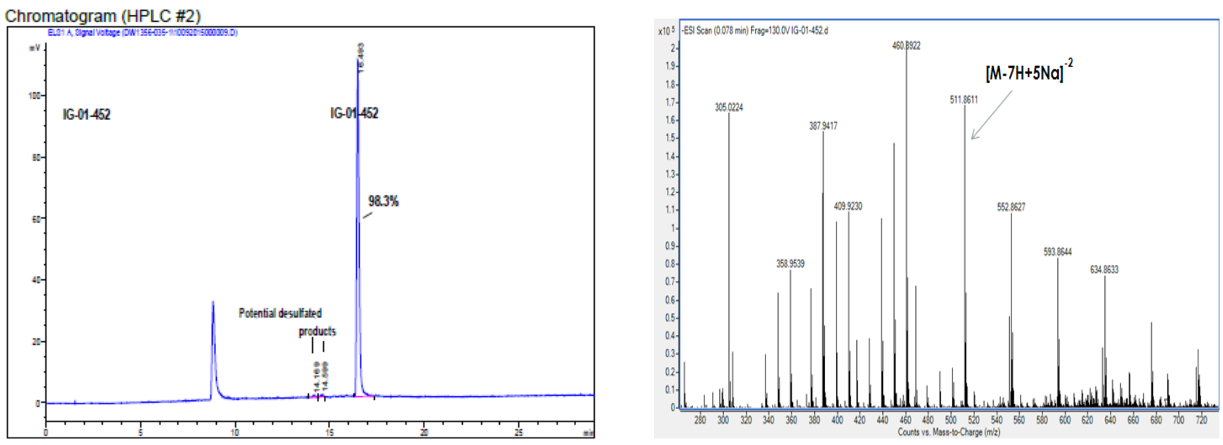

Methyl 2,3,4,6-tetra-*O*-sulfonato-β-D-glucopyranosyl-(1→4)-2,3,6-tri-*O*-sulfonato-β-D-glucopyranoside heptasodium salt (S4, mCBS)

Compound **S4** was prepared from D-cellobiose in 92% yield following **Procedure C**, **D**, **E** and **A**. **^{1}H NMR** (400 MHz, D_2O) δ 4.91 (d, J = 7.3 Hz, 1H, H-1), 4.86 (d, J = 6.2 Hz, 1H, H-1'), 4.76 – 4.67 (m, 2H, H-3', H-3), 4.63 (dd, J = 11.2, 3.2 Hz, 1H, H-6'a), 4.55 – 4.48 (m, 2H, H-3, H-6a), 4.46 (appt, J = 6.1 Hz, 1H, H-2'), 4.44 – 4.37 (m, 2H, H-2, H-6b), 4.27 (dd, J = 11.1, 7.1 Hz, 1H, H-6'b), 4.23

(appt, J = 7.6 Hz, 1H, H-4'), 4.11 – 4.03 (m, 2H, H-5, H-5'), 3.61 (s, 3H, OCH_3); **^{13}C NMR** (100 MHz, D_2O) δ 101.07 (C-1), 100.01 (C-1'), 77.76 (H-3'), 77.53 (C-3), 77.49 (C-2), 77.23 (C-2'), 74.42 (C-4'), 73.65 (C-4), 73.56 (C-5), 73.06 (C-5'), 67.78 (C-6'), 66.57 (C-6), 57.05 (OCH_3). ^{1}H NMR spectroscopic data are in agreement with published values[3]; **HRMS** (ESI) calculated for $C_{13}H_{22}O_{32}S_7$ $[M-2H]^{2-}$ m/z -456.9070, found -456.9076.

Supplementary Fig. 25 | ^{1}H-NMR spectrum of S4 (400 MHz, D_2O).

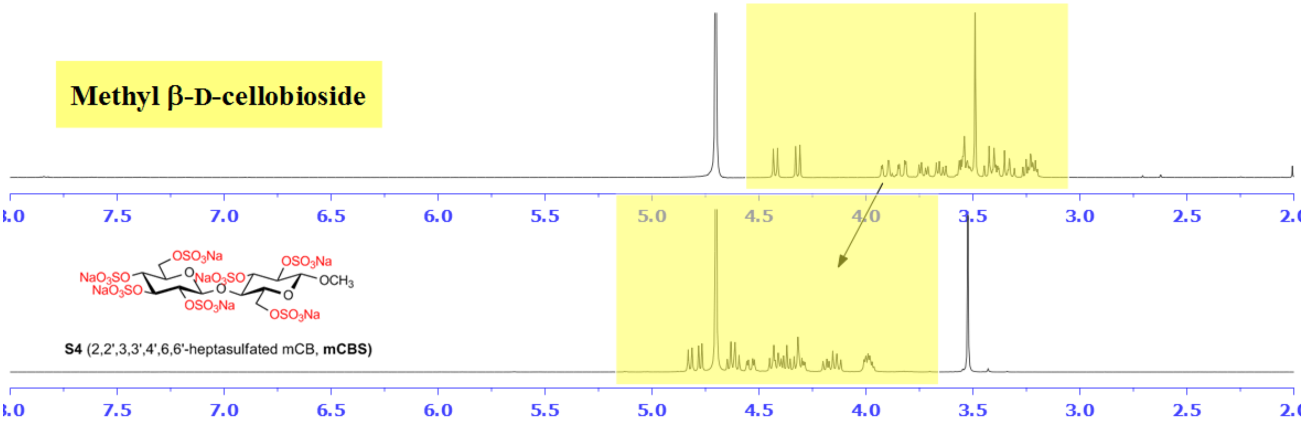

Supplementary Fig. 26 | ^{13}C-NMR spectrum of S4 (100 MHz, D_2O).

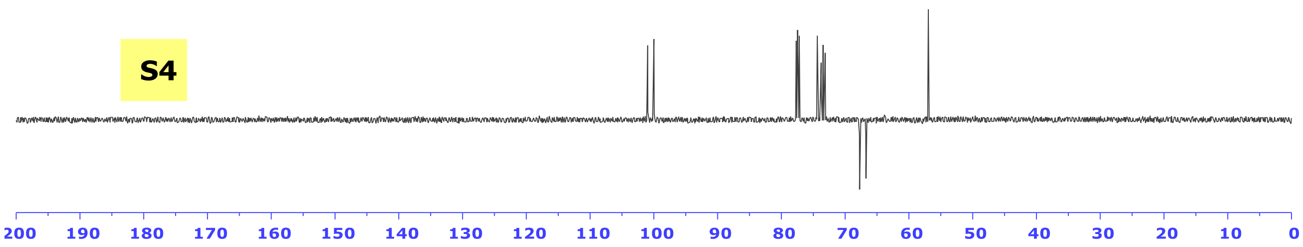

Supplementary Fig. 27 | 2D ^{1}H-^{1}H COSY and 2D ^{1}H-^{13}C HSQC spectrum of S4.

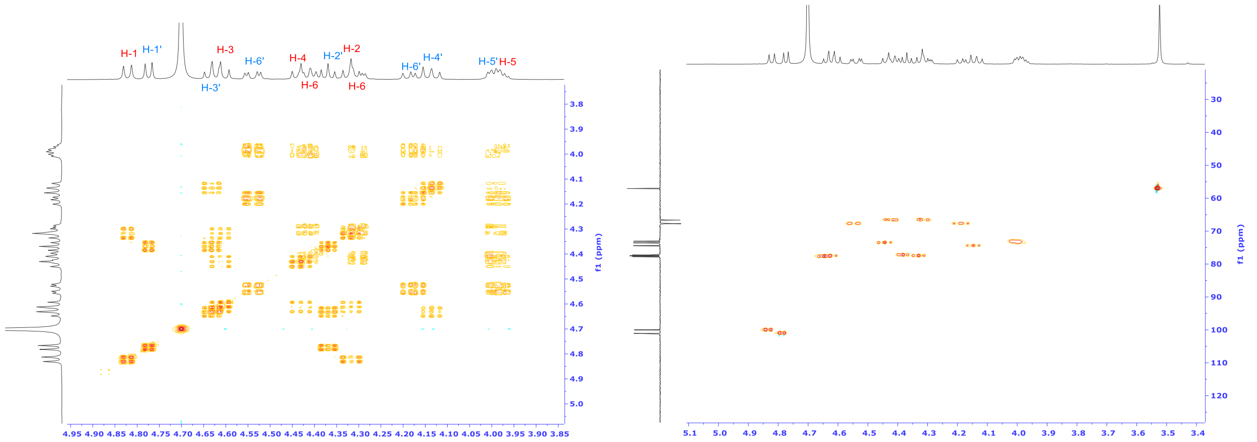

Supplementary Fig. 28 | HPLC analysis and HRMS spectrum of S4.

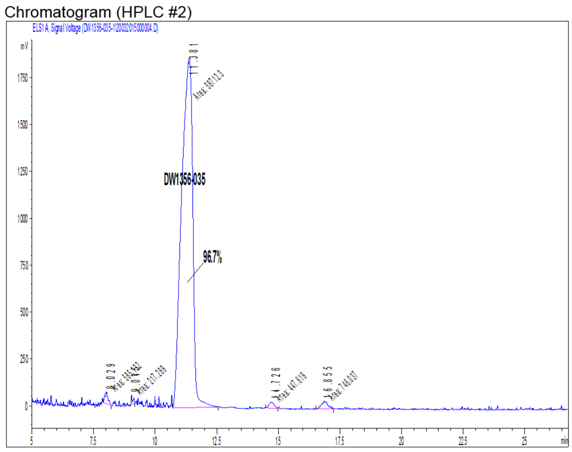

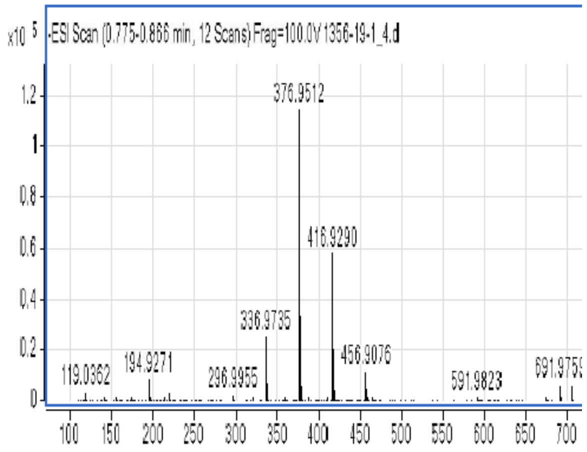

2,3,4,6-Tetra-*O*-sulfonato-β-D-galactopyranosyl-(1→4)-1,2,3,6-tetra-*O*-sulfonato-α,β-D-glucopyranose octasodium salt (S5)

Compound **S5** was prepared from D-lactose in 22 % yield following **Procedure A**. **^{1}H NMR** (400 MHz, D_2O) δ 6.06 (d, *J* = 3.5 Hz, 1H, H-1), 5.15 (d, *J* = 3.1 Hz, 1H, H-4'), 4.85 (d, *J* = 7.8 Hz, 1H, H-1'), 4.77 – 4.68 (m, 2H, H-6a, H-3), 4.58 (dd, *J* = 10.0, 3.1 Hz, 1H, H-3'), 4.54 – 4.44 (m, 2H, H-2, H-2'), 4.40 – 4.24 (m, 4H, H-2', H-6a', H-6b, H-6b', H-5), 4.20 – 4.10 (m, 2H, H-4, H-5'); **^{13}C NMR** (150 MHz, D_2O) δ 100.84 (C-1'), 95.15 (C-1), 75.78 (C-3'), 75.64 (C-2') , 75.40 (C-3), 75.32 (C-4'), 74.99 (C-4), 74.14 (C-2), 71.93 (C-5'), 70.36 (C-5), 66.82 (C-6'), 65.36 (C-6); **HRMS** (ESI) calculated for $C_{12}H_{22}O_{35}S_8$ [M-8H+6Na]$^{-2}$.

Supplementary Fig. 29 | ^{1}H-NMR spectrum of S5 (400 MHz, D_2O).

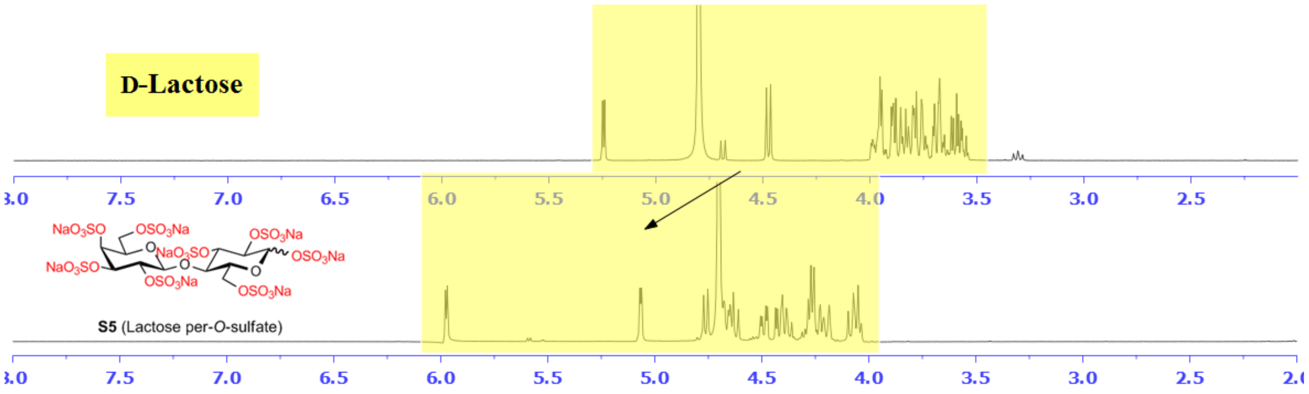

Supplementary Fig. 30 | ^{13}C-NMR spectrum of S5 (100 MHz, D_2O).

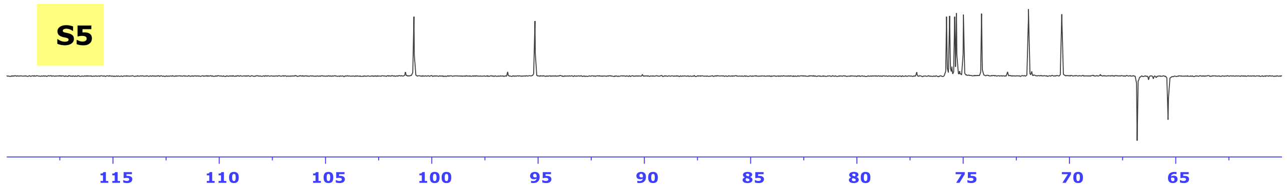

Supplementary Fig. 31 | 2D ^{1}H-^{1}H COSY and 2D ^{1}H-^{13}C HSQC spectrum of S5.

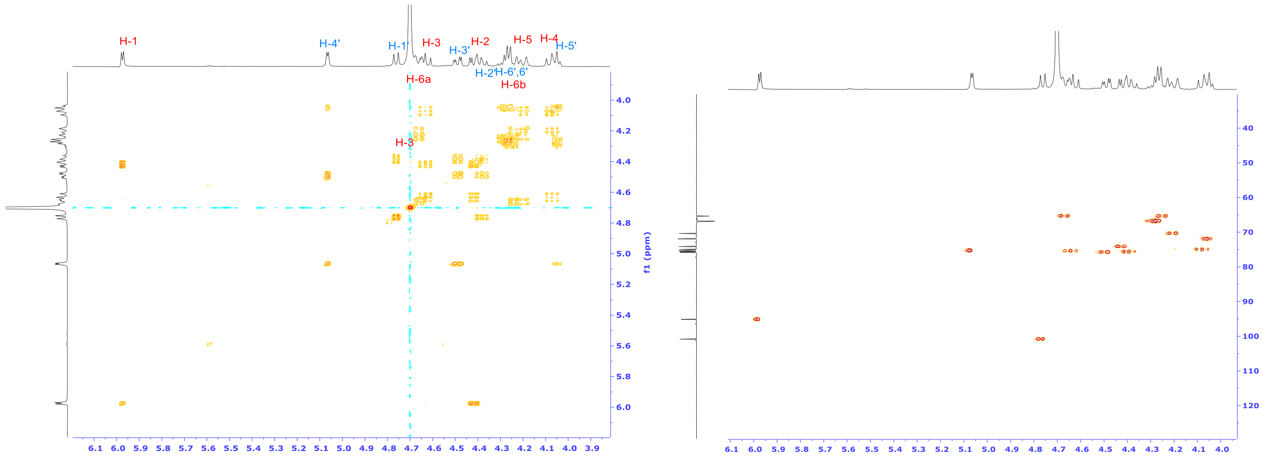

Supplementary Fig. 32 | HPLC analysis and HRMS spectrum of S5.

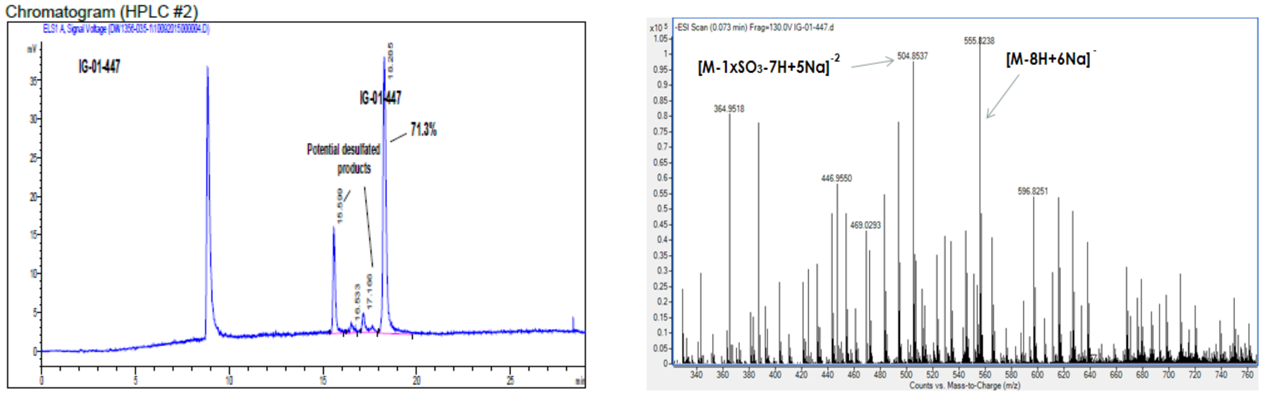

Methyl 2,3,4,6-tetra-*O*-sulfonato-β-D-galactopyranosyl-(1→4)-2,3,6-tetra-*O*-sulfonato-β-D-glucopyranoside heptasodium salt (S6)

Compound **S6** was prepared from D-lactose in 18% yield following **Procedure C**, **D**, **E** and **B**. **^{1}H NMR** (400 MHz, D_2O) δ 5.12 (d, *J* = 3.0 Hz, 1H, H-4'), 4.89 (d, *J* = 5.6 Hz, 1H, H-1), 4.82 – 4.76 (m, 1H, H-1'), 4.70 (dd, *J* = 6.4, 4.9 Hz, 1H, H-3), 4.56 – 4.43 (m, 3H, H-3', H-6a, H-2), 4.41 (dd, *J* = 10.0, 7.7 Hz, 1H, H-2'), 4.35 – 4.28 (m, 3H, H-6b, H-6'a, H-6'b), 4.21 (appt, *J* = 7.1 Hz, 1H, H-4), 4.14 – 4.05 (m, 2H, H-5', H-5), 3.56 (s, 3H, OCH_3); **^{13}C NMR** (151 MHz, D_2O) δ 100.98 (C-1'), 100.66 (C-1), 77.72 (C-3), 77.09 (C-2), 75.72 (C-3'), 75.44 (C-2'), 75.11 (C-4'), 75.02 (C-4), 73.10

(C-5), 71.81 (C-5'), 66.58 (C-6), 66.36 (C-6'), 56.79 (OCH_3); **HRMS** (ESI) calculated for $C_{13}H_{24}O_{32}S_7$ $[M-7H+5Na]^{-2}$ m/z -511.8618, found -511.8607.

Supplementary Fig. 33 | ^{1}H-NMR spectrum of **S6** (400 MHz, D_2O).

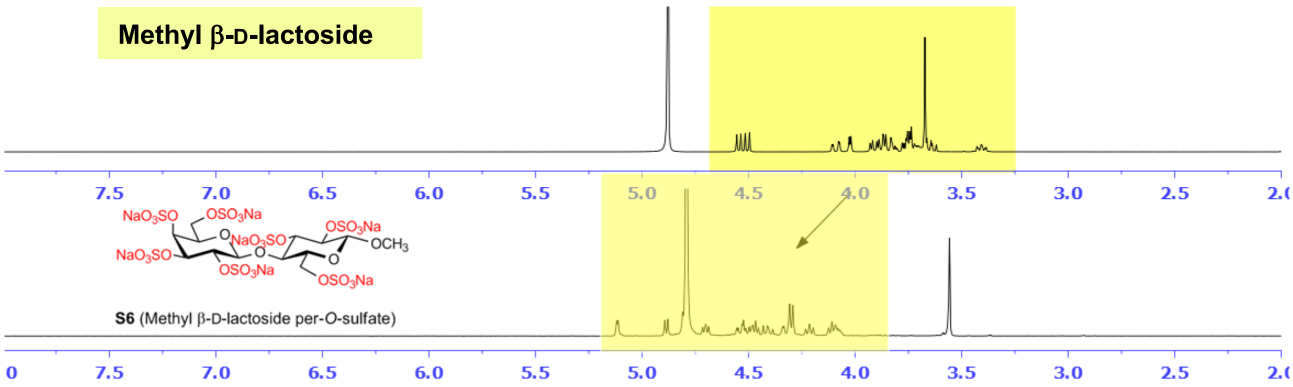

Supplementary Fig. 34 | ^{13}**C-NMR spectrum of S6 (100 MHz,** D_2O**).**

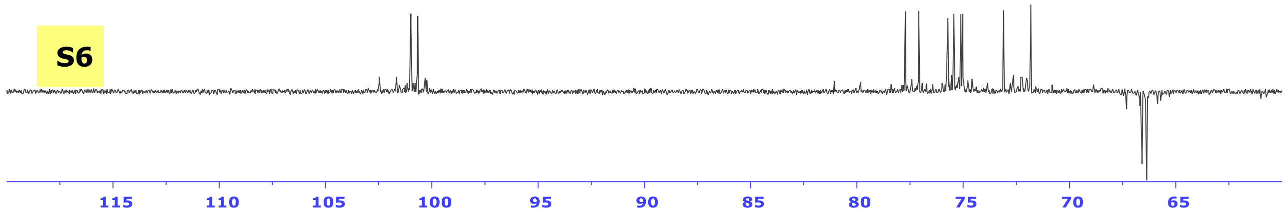

Supplementary Fig. 35 | 2D ^{1}**H-**^{1}**H COSY and 2D** ^{1}**H-**^{13}**C HSQC spectrum of S6.**

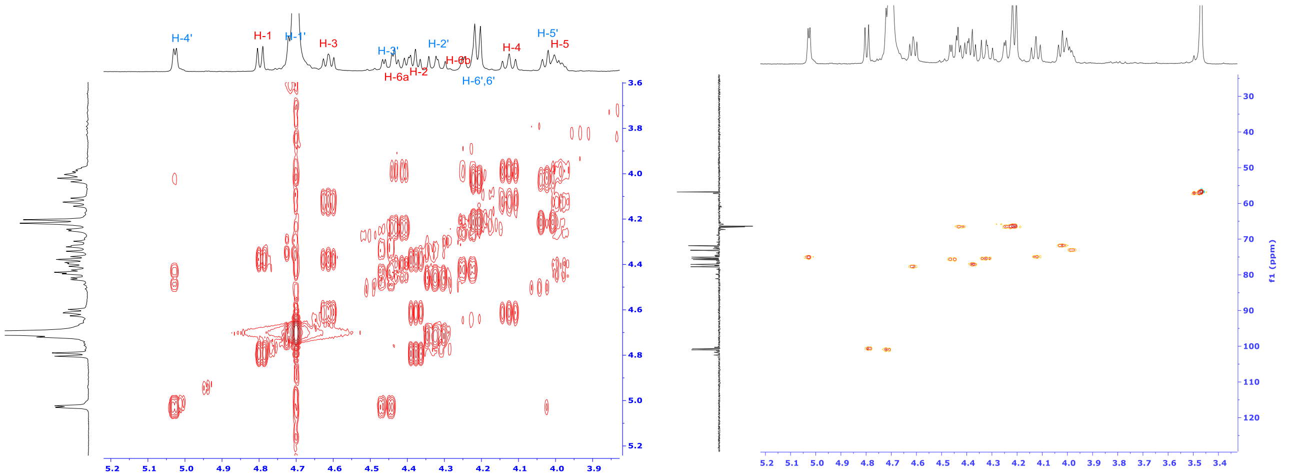

Supplementary Fig. 36 | HPLC analysis and HRMS spectrum of S6.

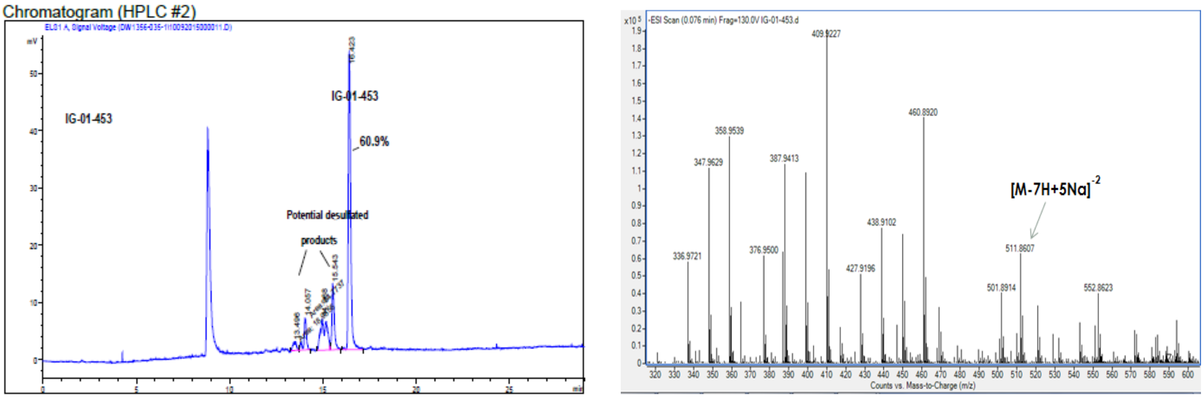

2,3,4,6-Tetra-*O*-sulfonato-β-D-galactopyranosyl-(1→4)-1,2,3,6-tetra-*O*-sulfonato-D-fructofuranose octasodium salt (S7)

Compound **S7** was prepared from D-lactulose in 26% yield following **Procedure B**. **^{1}H NMR** (400 MHz, D_2O) δ 5.30 (d, *J* = 1.7 Hz, 1H, H-3), 5.24 (d, *J* = 18.2 Hz, 1H, H-1a), 5.08 (d, *J* = 3.2 Hz, 1H, H-4'), 5.05 (d, *J* = 18.2 Hz, 1H, H-1b), 4.88 (d, *J* = 8.0 Hz, 1H, H-1'), 4.83 – 4.75 (m, 1H, H-5), 4.71 – 4.63 (m, 2H, H-6a, H-4), 4.62 – 4.50 (m, 2H, H-6b, H-3'), 4.40 (dd, *J* = 10.0, 7.8 Hz, 2H, H-2'), 4.36 – 4.26 (m, 2H, H-6a'), 4.24 – 4.14 (m, 2H, H-6b'), 4.10 (appt, *J* = 6.2 Hz, 1H, H-5'); **^{13}C NMR** (100 MHz, D_2O) δ 203.65 (C-2), 100.76 (C-1'), 79.76 (C-3), 76.22 (C-5), 75.59 (C-4), 75.52 (C-3'), 75.04 (C-2'), 74.95 (C-4'), 71.91 (C-5'), 71.24 (C-1), 66.27 (C-6'), 65.27 (C-6); **HRMS** (ESI) calculated for $C_{12}H_{22}O_{35}S_8$ [M-8H+6Na]$^{-2}$ m/z -555.8233, found -555.8232.

Supplementary Fig. 37 | ^{1}H-NMR spectrum of S7 (400 MHz, D_2O).

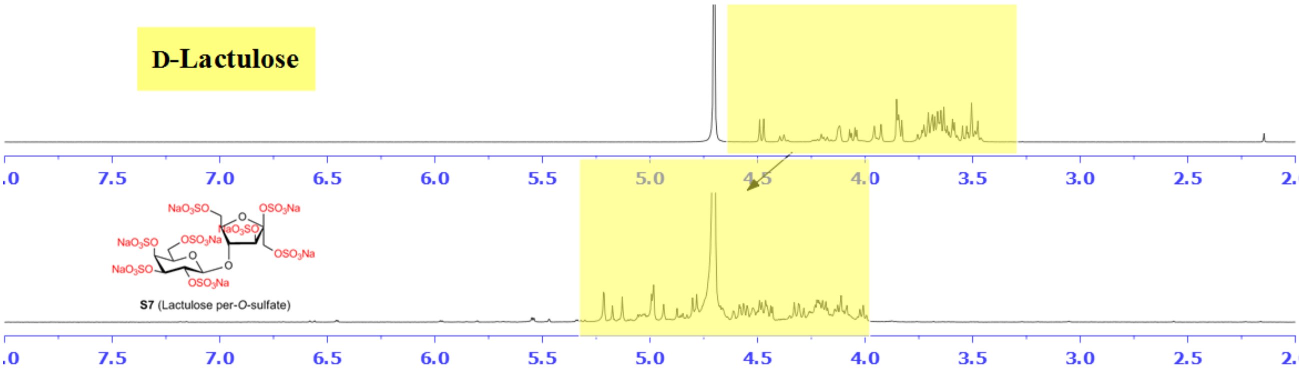

Supplementary Fig. 38 | ^{13}C-NMR spectrum of S7 (100 MHz, D_2O).

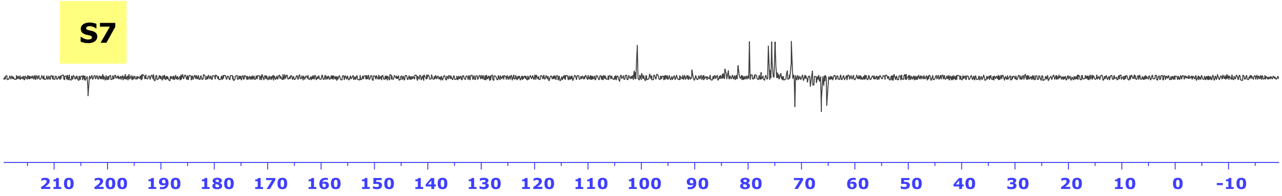

Supplementary Fig. 39 | 2D ^{1}H-^{1}H COSY and 2D ^{1}H-^{13}C HSQC spectrum of S7.

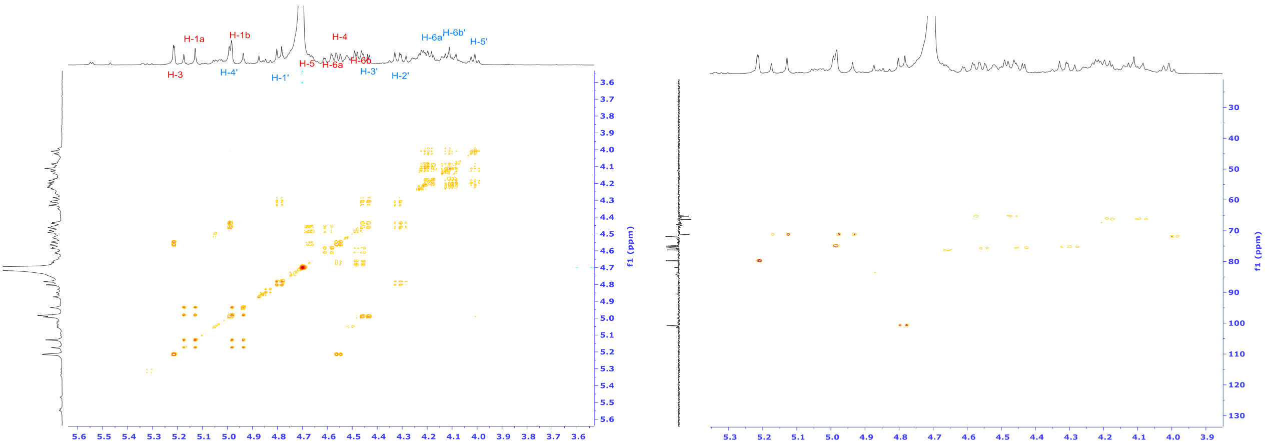

Supplementary Fig. 40 | HPLC analysis and HRMS spectrum of S7.

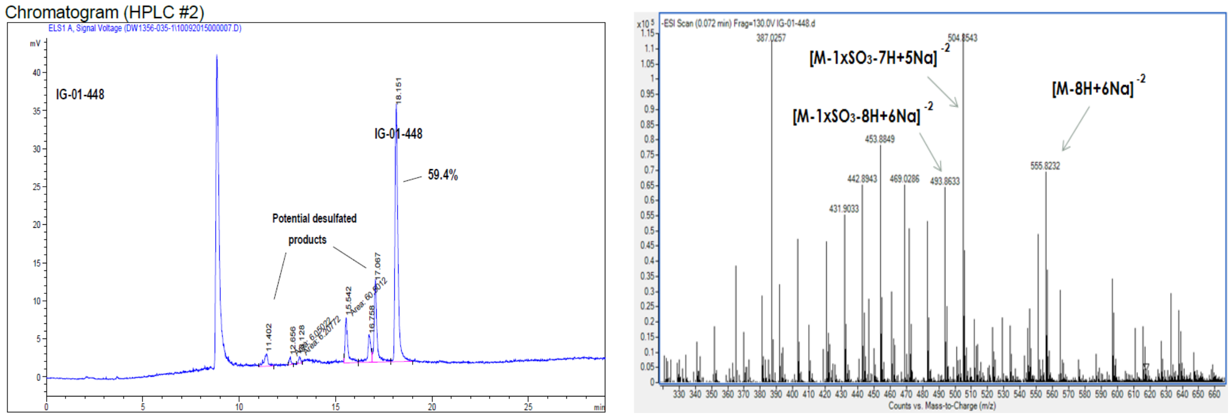

2,3,4-Tri-*O*-sulfonato-β-D-xylopyranosyl-(1→4)-1,2,3-tri-*O*-sulfonato-α,β-D-xylopyranose hexasodium salt (S8)

Compound **S8** was prepared from D-xylobiose in 66% yield following **Procedure A**. **^{1}H NMR** (400 MHz, D_2O) δ 5.99 (d, J = 3.3 Hz, 0.4H, H-1β), 5.84 (s, 0.6H, H-1α), 5.23 (s, 0.6H, H-1'), 5.16 (s, 0.4H, H-1'), 4.92 – 4.48 (m, 5.2H), 4.45 (dd, J = 9.3, 3.4 Hz, 0.4H), 4.35 (d, J = 11.8 Hz, 0.6H, H-5a), 4.10 – 3.85 (m, 3.2H); **^{13}C NMR** (100 MHz, D_2O) δ 99.69 (C-1'), 97.63 (C-1'), 95.53, 95.37 (C-1β), 75.65 (C-1α), 74.95, 74.51, 74.38, 73.67, 72.39, 71.88, 71.43, 71.29, 71.21, 71.08, 70.81, 60.85 (C-6), 59.14 (C-6), 58.59 (C-6), 58.47 (C-6); **HRMS** (ESI) calculated for $C_{10}H_{18}O_{27}S_6$ [M-1xSO^3-5H+3Na]$^{-2}$ m/z -372.9047, found -372.9050; [M-2xSO^3-4H+2Na]$^{-2}$ m/z -321.9353, found -321.9360.

Supplementary Fig. 41 | ^{1}H-NMR spectrum of S8 (400 MHz, D_2O).

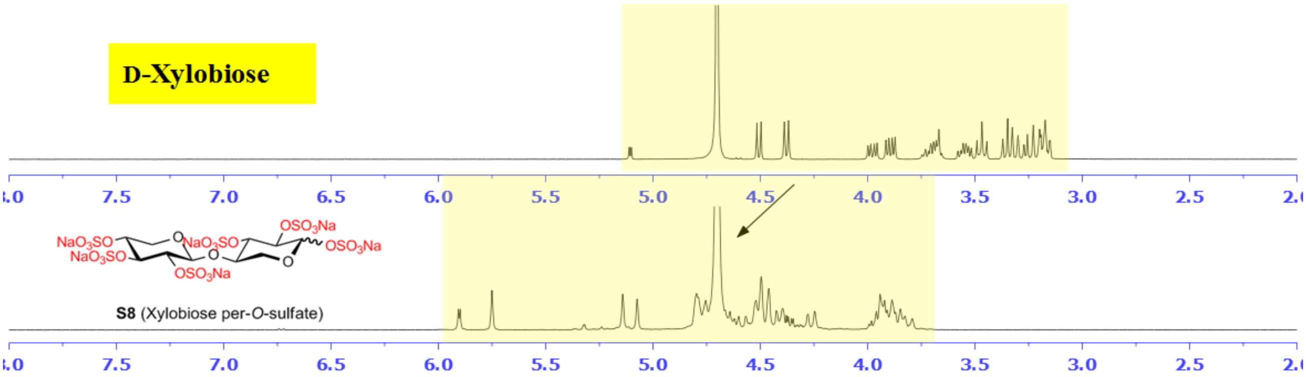

Supplementary Fig. 42 | ^{13}C-NMR spectrum of S8 (100 MHz, D_2O).

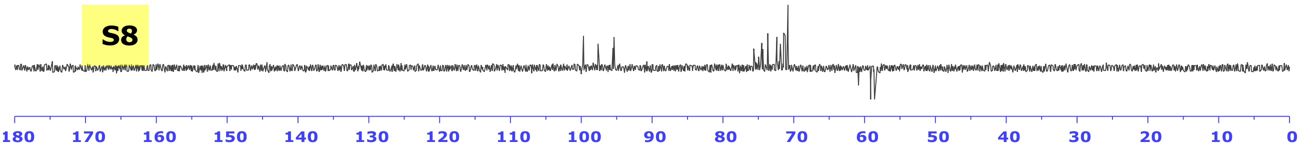

Supplementary Fig. 43 | 2D ^{1}H-^{1}H COSY and 2D ^{1}H-^{13}C HSQC spectrum of S8.

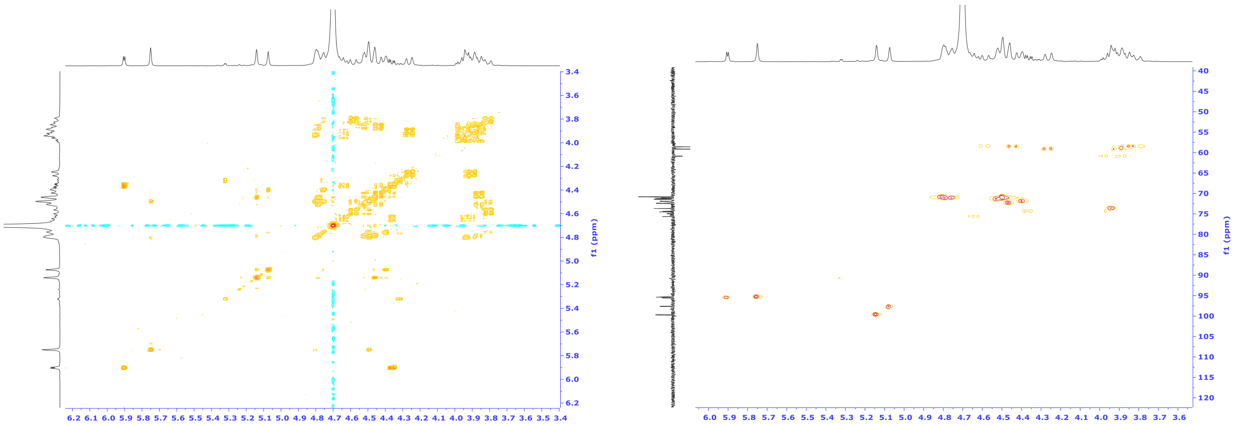

Supplementary Fig. 44 | HPLC analysis and HRMS spectrum of S8.

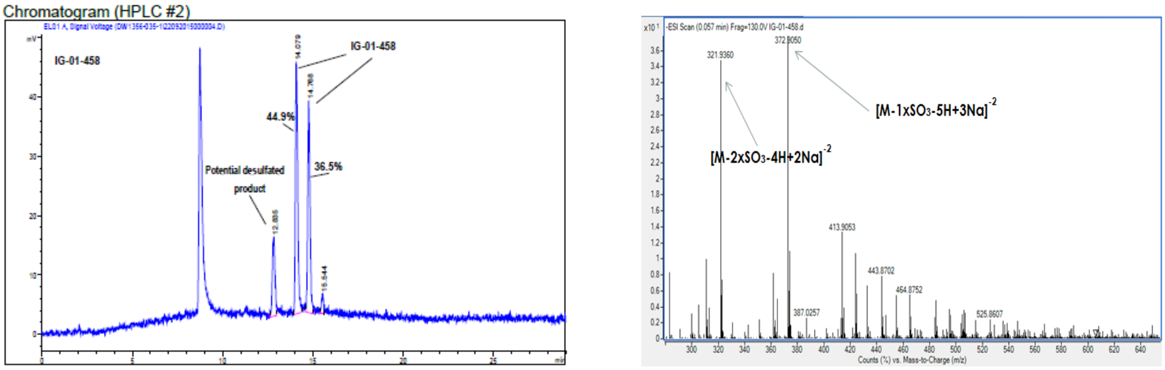

Methyl 2,3,4-tri-*O*-sulfonato-β-D-xylopyranosyl-(1→4)-2,3-di-*O*-sulfonato-β-D-xylopyranoside pentasodium salt (S9)

Compound **S9** was prepared from D-xylobiose in 47% yield following **Procedure C**, **D**, **E** and **A**. ^{1}**H NMR** (400 MHz, D_2O) δ 5.18 (s, 1H, H-1'), 4.90 (s, 1H , H-1), 4.85 – 4.81 (m, 2H, H-3', H-3), 4.58 – 4.54 (m, 1H, H-4'), 4.54 – 4.49 (m, 2H, H-5a', H-2'), 4.41 (s, 1H, H-2), 4.13 (dd, J = 13.2, 2.0 Hz, 1H, H-5a), 3.96 –3.80 (m, 3H, H-4, H-5b', H-5b), 3.46 (s, 3H); ^{13}**C NMR** (100 MHz, D_2O) δ 99.49 (C-1'), 98.54 (C-1), 74.15 (C-4), 72.34 (C-2'), 71.82 (C-2), 71.80 (C-3), 71.40 (C-4'), 71.16 (C-3'), 58.55 (C-5'), 58.00 (C-5), 55.55 (OCH_3); **HRMS** (ESI) calculated for $C_{11}H_{20}O_{24}S_5$ $[M-5H+3Na]^{-2}$ m/z -379.9125, found -379.9140.

Supplementary Fig. 45 | ^{1}H-NMR spectrum of S9 (400 MHz, D_2O).

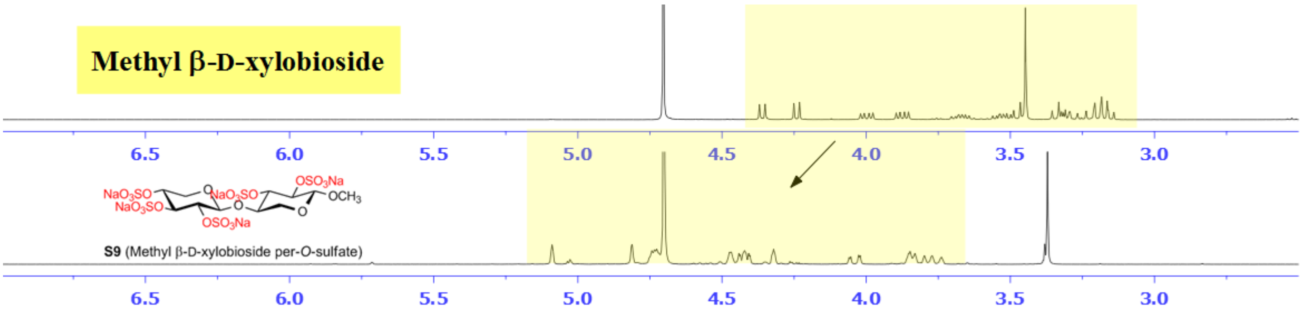

Supplementary Fig. 46 | ^{13}C-NMR spectrum of S9 (100 MHz, D_2O).

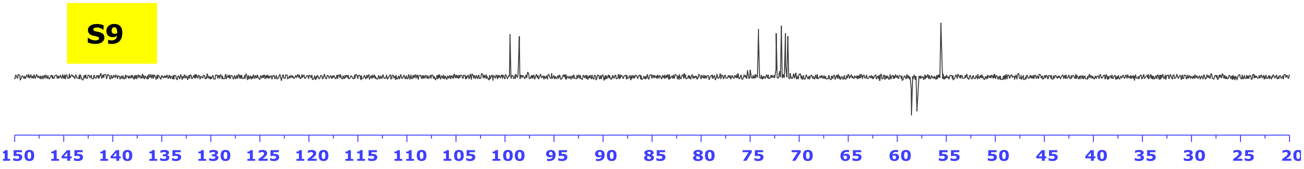

Supplementary Fig. 47 | 2D ^{1}H-^{1}H COSY and 2D ^{1}H-^{13}C HSQC spectrum of S9.

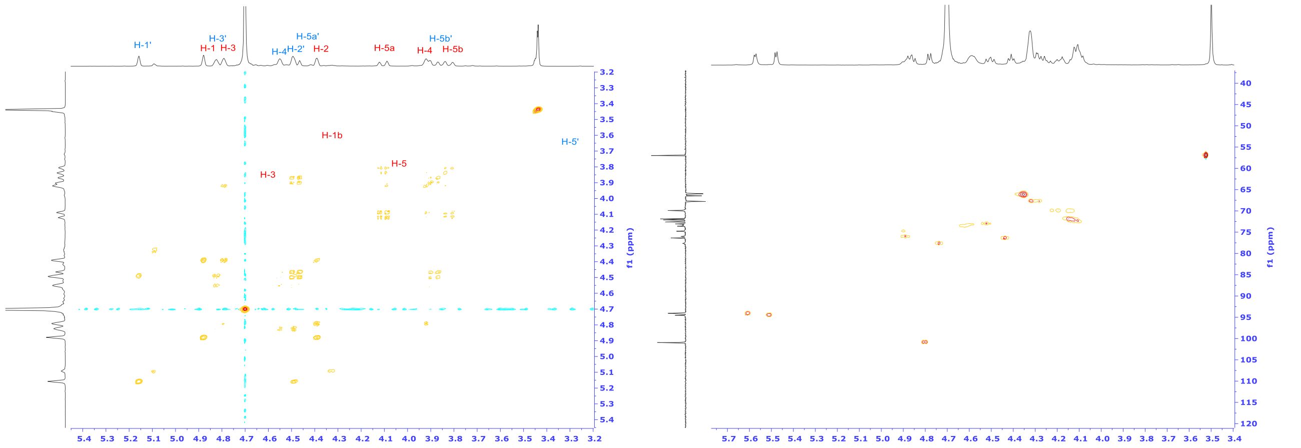

Supplementary Fig. 48 | HPLC analysis and HRMS spectrum of S9.

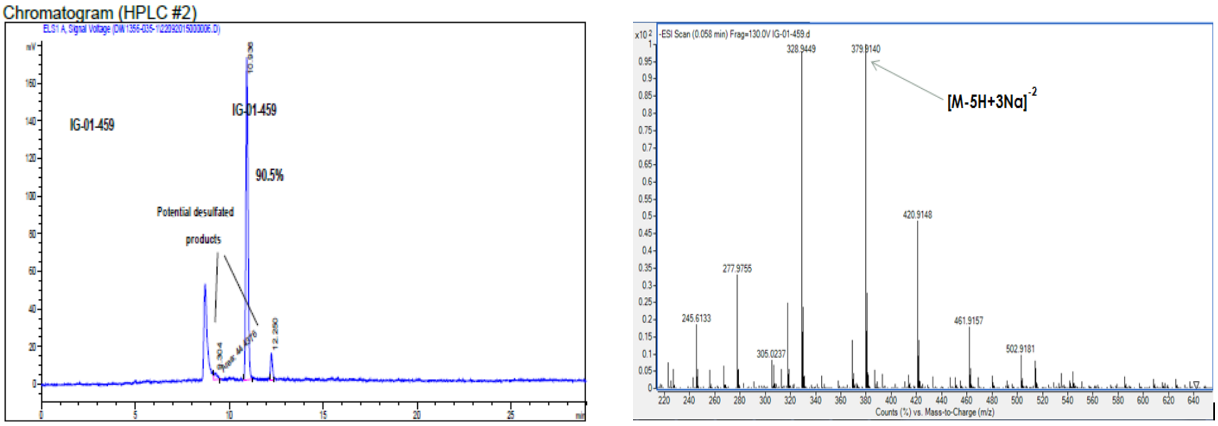

Methyl 2,3,4,6-tetra-*O*-sulfonato-α-D-glucopyranosyl-(1→4)-2,3,6-tri-*O*-sulfonato-α-D -glucopyranosyl-(1→4)-2,3,6-tri-*O*-sulfonato-β-D-glucopyranoside decasodium salt (S10)

Compound **S10** was prepared from D-maltotriose in 10% yield following **Procedure C**, **D**, **E** and **B**. ^{1}**H NMR** (400 MHz, D_2O) δ 5.66 (d, *J* = 3.4 Hz, 1H, H-1''), 5.57 (d, *J* = 3.6 Hz, 1H, H-1'), 5.01 – 4.91 (m, 2H, H-3', H-3''), 4.87 (d, *J* = 5.2 Hz, 1H, H-1), 4.83 – 4.75 (m, 1H, H-3), 4.71 – 4.65 (m, 2H, H-2', H-2''), 4.59 (dd, *J* = 8.0, 3.6 Hz, 1H, H-4*), 4.50 (appt, *J* = 5.1 Hz, 1H, H-2), 4.45 – 4.31 (m, 6H, 6 x H-6*), 4.31 – 4.24 (m, 1H, H-4*), 4.25 – 4.12 (m, 4H, H-4*, H-5, H-5', H-5''), 3.59 (s, 3H, OCH_3); ^{13}**C NMR** (100 MHz, D_2O) δ 100.94 (C-1), 94.52 (C-1'), 94.07 (C-1''), 77.60 (C-3), 76.36 (C-2), 76.00 (C-3'), 74.77 (C-3''), 73.58 (C-2'), 73.34 (C-2''), 73.06 (C-4*), 72.60 (C-5*), 72.12 (C-5*), 71.89 (C-5*), 69.90 (C-5*), 69.90 (C-4*), 67.79 (C-6*), 66.47 (C-6*), 65.97 (C-6*), 56.99 (OCH_3). Note*: Due to the proton NMR spectrum complexity, complete spectrum assignment was limited; **HRMS** (ESI) calculated for $C_{19}H_{34}O_{46}S_{10}$ $[M-10H+8Na]^{-2}$ m/z -745.7963, found -745.7949; $[M-1xSO_3-9H+7Na]^{-2}$ m/z -694.8270, found -694.8266.

Supplementary Fig. 49 | ^{1}H-NMR spectrum of S10 (400 MHz, D_2O).

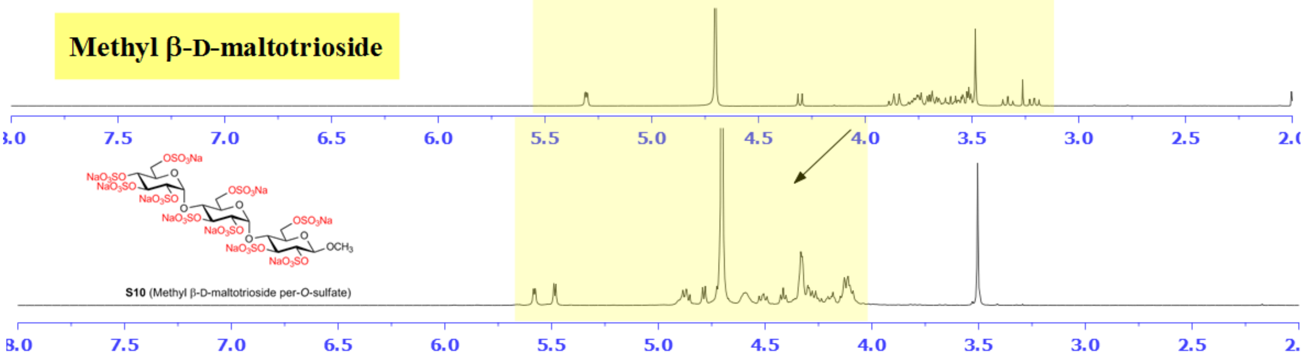

Supplementary Fig. 50 | ^{13}C-NMR spectrum of S10 (100 MHz, D_2O).

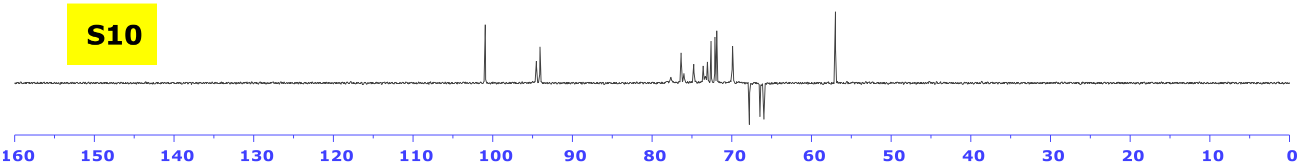

Supplementary Fig. 51 | 2D ^{1}H-^{1}H COSY and 2D ^{1}H-^{13}C HSQC spectrum of S10.

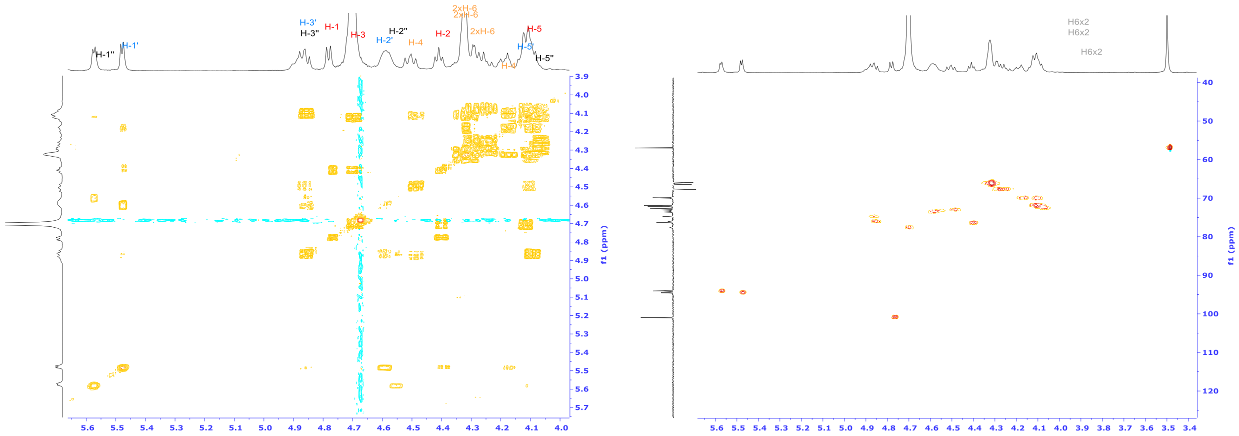

Supplementary Fig. 52 | HPLC analysis and HRMS spectrum of S10.

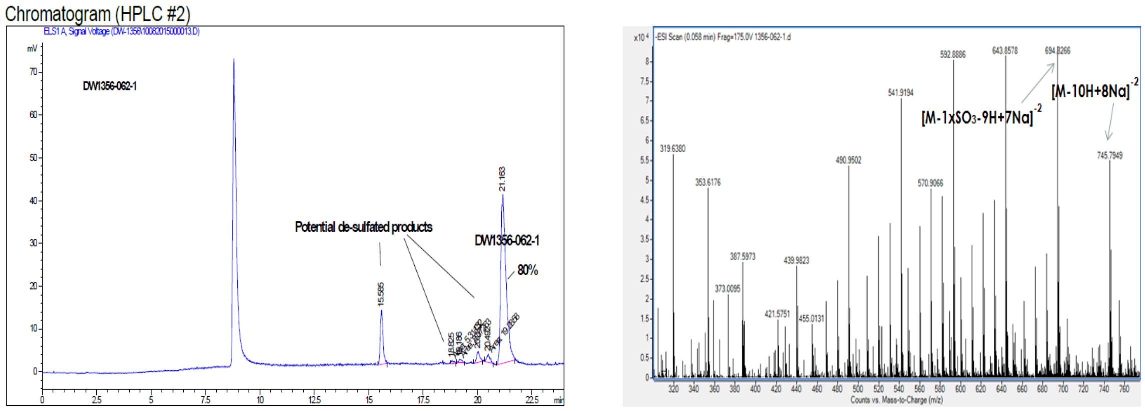

2,3,4,6-Tetra-*O*-sulfonato-α-D-glucopyranosyl-(1→3)-1,4,6-tri-*O*-sulfonato-β-D-fructofuranosyl-(2→1)-2,3,4,6-tetra-*O*-sulfonato-α-D-glucopyranoside undecasodium salt (S11)

Compound **S11** was prepared from D-melezitose in 66% yield following **Procedure B**.

^{1}H NMR (400 MHz, D_2O) δ 5.89 (d, J = 3.8 Hz, 1H, H-1*), 5.76 (d, J = 3.7 Hz, 1H, H-1*), 4.90 – 4.83 (m, 2H)), 4.82 – 4.72 (m, 3H), 4.64 – 4.37 (m, 11H), 4.29 (s, 2H, H-1'a, H-1'b), 4.14 (d, J = 9.9 Hz, 1H, H-3'); **^{13}C NMR** (100 MHz, D_2O) δ 102.62 (C-2'), 95.91 (C-1*), 89.40 (C-1*), 79.50, 78.92, 77.88, 75.81, 75.42, 74.39, 74.11, 73.84, 73.41, 69.38 (C-6'), 68.83 (C-3'), 68.79, 66.25 (C-6*), 65.97 (C-6*), 65.82 (C-1'). Note*: Due to the proton NMR spectrum complexity, complete spectrum

assignment was limited; **HRMS** (ESI) calculated for $C_{18}H_{32}O_{49}S_{11}$ [M-11H+9Na]$^-$ m/z -789.7579, found -789.7553.

Supplementary Fig. 53 | ^{1}H-NMR spectrum of S11 (400 MHz, D$_2$O).

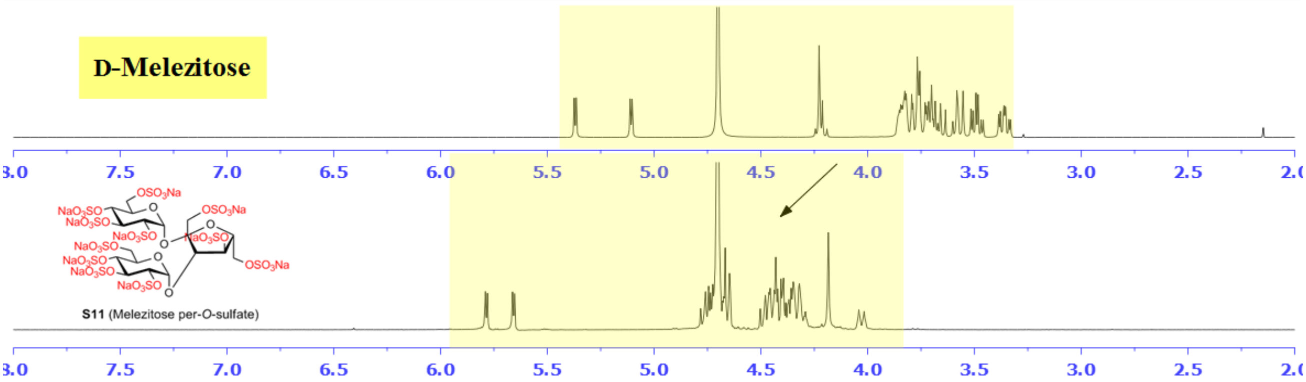

Supplementary Fig. 54 | ^{13}C-NMR spectrum of S11 (100 MHz, D$_2$O).

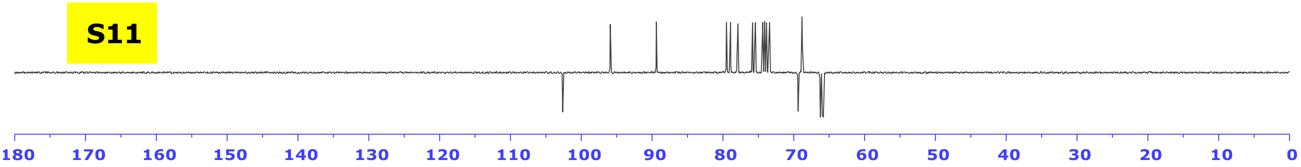

Supplementary Fig. 55 | 2D ^{1}H-^{1}H COSY and 2D ^{1}H-^{13}C HSQC spectrum of S11.

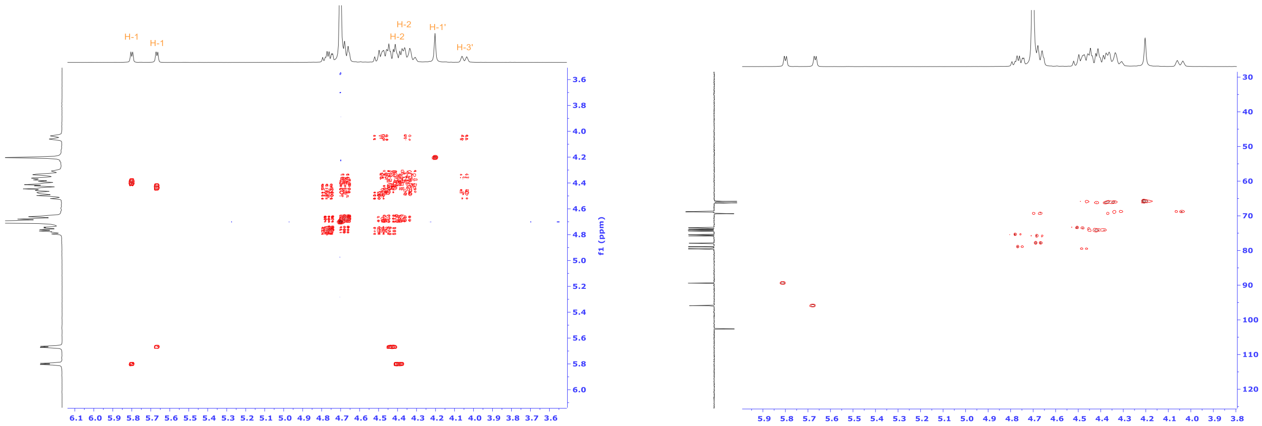

Supplementary Fig. 56 | HPLC analysis and HRMS spectrum of S11.

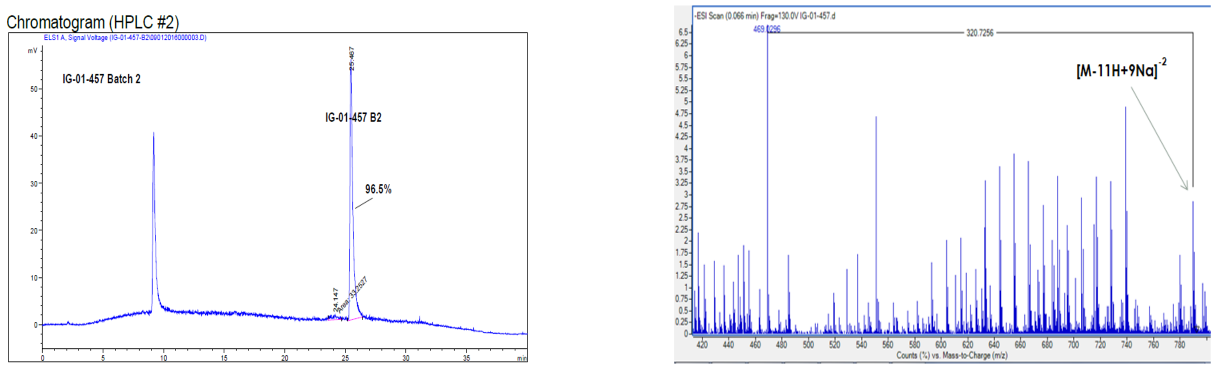

Methyl 4,6-di-*O*-sulfonato-β-D-glucopyranosyl-(1→4)-β-D-glucopyranoside disodium salt (S12)

Compound **S12** was prepared from methyl β-D-cellobioside in 50% yield following procedures described in **Supplementary Scheme 4**. **^{1}H NMR** (600 MHz, D_2O) δ 4.58 (d, *J* = 8.0 Hz, 1H, H-1'), 4.50 (dd, *J* = 11.4, 2.2 Hz, 1H, H-6'a), 4.42 (d, *J* = 8.0 Hz, 1H, H-1), 4.16 (m, 2H, H-4', H-6'b), 4.00 (dd, *J* = 12.3, 1.4 Hz, 1H, H-6a), 3.90 (ddd, *J* = 9.9, 7.4, 2.3 Hz, 1H, H-5'), 3.80 – 3.84 (m, 1H, H-6b), 3.76 (appt, *J* = 9.3 Hz, 1H, H-3'), 3.62 – 3.68 (m, 3H, H-3, H-5, H-4), 3.58 (s, 3H, OCH_3), 3.44 (dd, *J* = 9.6, 8.0 Hz, 1H, H-2'), 3.33 (appt, *J* = 8.5 Hz, 1H, H-2). **^{13}C NMR** (150 MHz, D_2O) δ 102.92 (C-1), 102.36 (C-1'), 79.56 (C-4), 76.47 (C-4'), 74.63 (C-5), 74.29 (C-3), 73.90 (C-3'), 72.75 (C-2), 72.72 (C-2'), 72.20 (C-5'), 67.25 (C-6'), 60.14 (C-6), 57.15 (OCH_3); **HRMS** (ESI) calculated for: $C_{13}H_{24}O_{17}S_2$ $[M–2H+Na]^{–}$ m/z 537.0241, found 537.0197; $C_{13}H_{24}O_{17}S_2$ $[M–2H]^{2–}$ m/z 257.0172, found 257.0153.

Supplementary Fig. 57 | ^{1}H-NMR spectrum of S12 (600 MHz, D_2O).

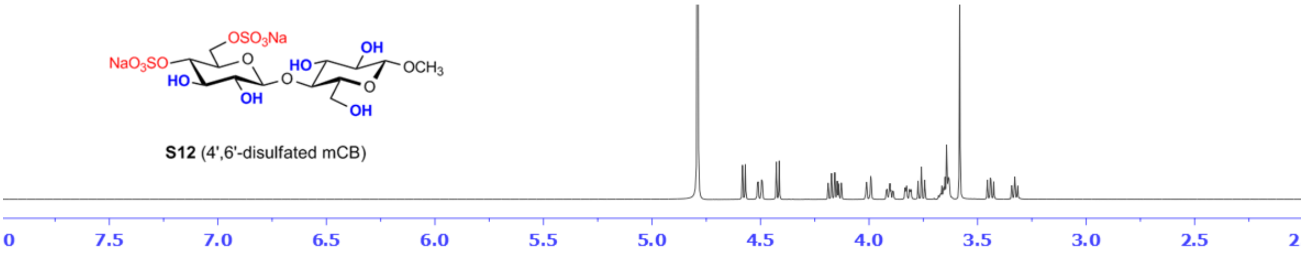

Supplementary Fig. 58 | ^{13}C-NMR spectrum of S12 (150 MHz, D_2O).

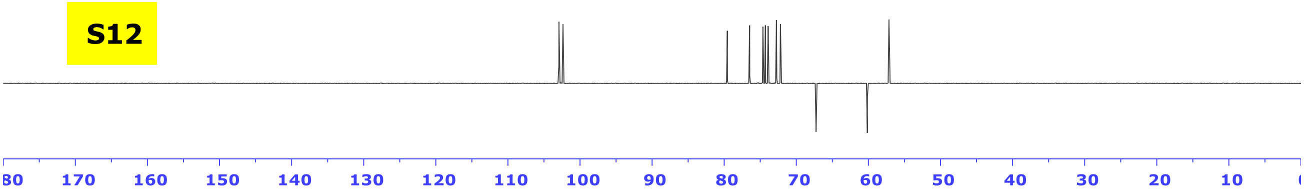

Supplementary Fig. 59 | 2D ^{1}H-^{1}H COSY and 2D ^{1}H-^{13}C HSQC spectrum of S12.

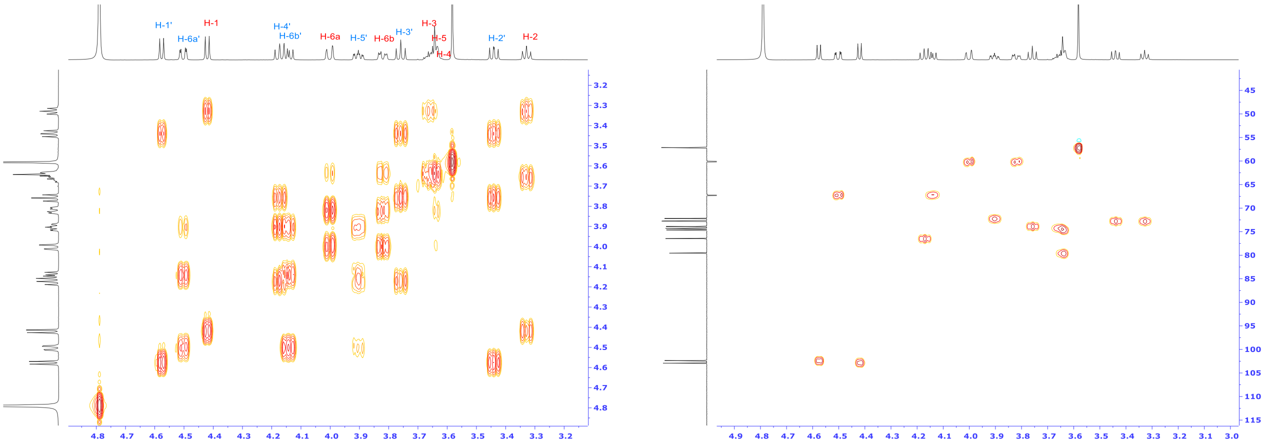

Supplementary Fig. 60 | HPLC analysis and HRMS spectrum of S12.

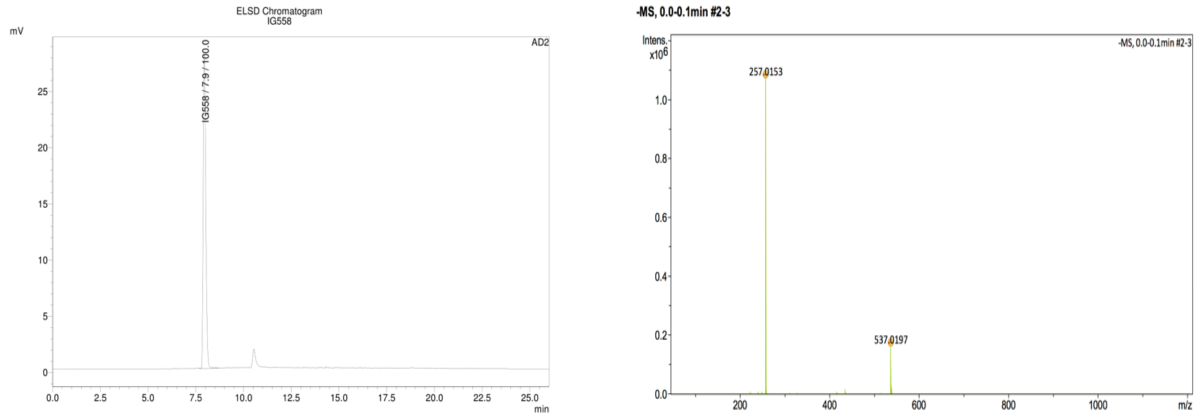

Methyl 4,6-di-*O*-sulfonato-β-D-glucopyranosyl-(1→4)-6-*O*-sulfonato-β-D-glucopyranoside trisodium salt (S13)

Compound **S13** was prepared from methyl β-D-cellobioside in 44% yield following procedures described in **Supplementary Scheme 4**. **^{1}H NMR** (600 MHz, D_2O) δ 4.62 (d, *J* = 7.9 Hz, 1H, H-1'), 4.50 (dd, *J* = 10.8, 1.8 Hz, 1H, H-H-6'a), 4.50 (d, *J* = 7.8 Hz, 1H, H-1), 4.43 (dd, *J* = 10.8, 3.0 Hz, 1H, H-H-6a), 4.32 (dd, *J* = 11.1, 4.9 Hz, 1H, H-6b), 4.22 – 4.13 (m, 1H, H-4', H-6'b), 3.94 – 3.89 (m, 1H, H-5'), 3.85 (ddd, *J* = 9.7, 5.0, 1.9 Hz, 1H, H-5), 3.77 (appt, *J* = 9.3 Hz, 1H, H-3'), 3.71 (appt, *J* = 9.1 Hz, 1H, H-4), 3.67 (appt, *J* = 8.9 Hz, 1H, H-3), 3.59 (s, 3H, OCH_3), 3.43 (appt, *J* = 9.2 Hz, 1H, H-2'), 3.35 (appt, *J* = 8.5 Hz, 1H, H-2); **^{13}C NMR** (150 MHz, D_2O) δ 102.99 (C-1), 102.30 (C-1'), 79.01 (C-4), 76.47 (C-4'), 74.24 (C-3), 73.87 (C-3'), 72.76 (C-2'), 72.63 (C-2), 72.44 (C-5), 72.14 (C-5'), 67.15 (C-6'), 66.38 (C-6), 57.25 (OCH_3); **HRMS** (ESI) calculated for: $C_{13}H_{24}O_{20}S_3$ $[M{-}3H{+}Na]^{2-}$ m/z 307.9831, found 307.9844; $C_{13}H_{24}O_{17}S_2$ $[M{-}3H]^{3-}$ m/z 197.6588, found 197.6598.

Supplementary Fig. 61 | ^{1}H-NMR spectrum of S13 (600 MHz, D_2O).

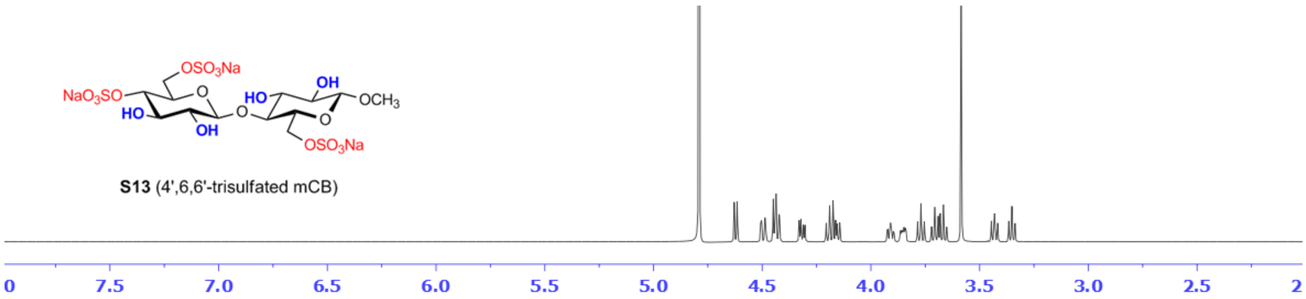

Supplementary Fig. 62 | ^{13}C-NMR spectrum of S13 (150 MHz, D_2O).

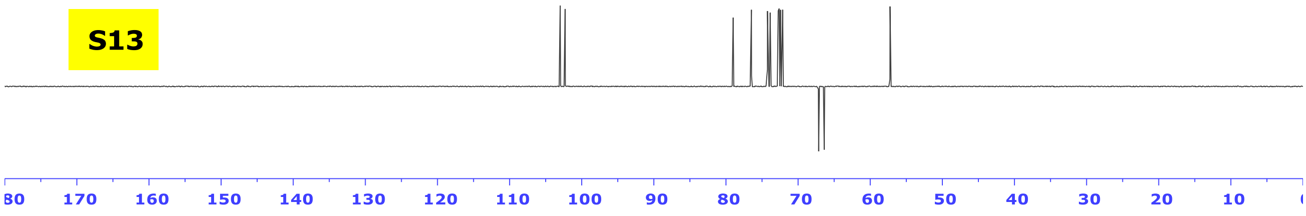

Supplementary Fig. 63 |. 2D ^{1}H-^{1}H COSY and 2D ^{1}H-^{13}C HSQC spectrum of S13.

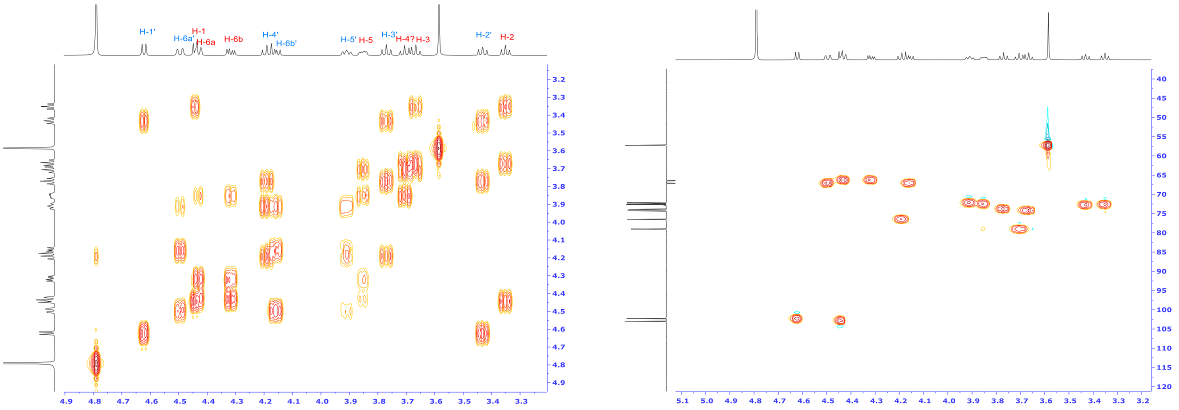

Supplementary Fig. 64 | HPLC analysis and HRMS spectrum of S13.

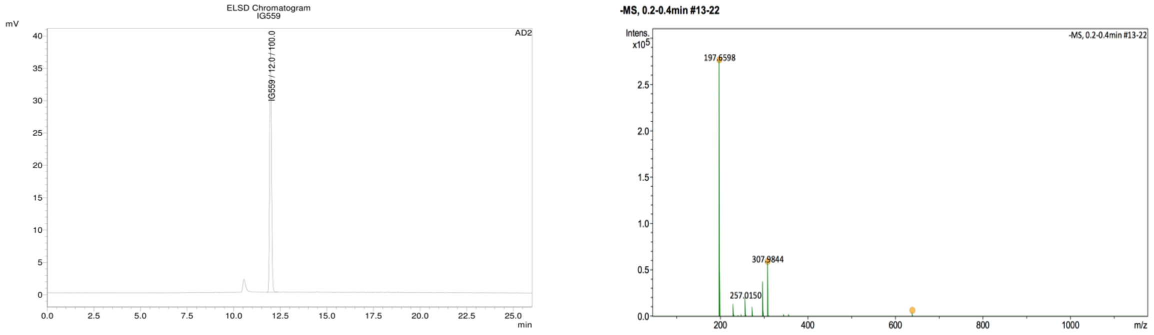

Methyl 2,3-di-*O*-sulfonato-β-D-glucopyranosyl-(1→4)-2,3-di-*O*-sulfonato-β-D-glucopyranoside tetrasodium salt (S14)

Compound **S14** was prepared from methyl β-D-cellobioside in 7% yield following procedures described in **Supplementary Scheme 4**. **^{1}H NMR** (600 MHz, D_2O) δ 4.86 – 4.74 (obscured, 3H, H-3', H-1', H-1), 4.42 (appt, J = 9.1 Hz, 1H, H-3), 4.36 (appt, J = 5.7 Hz, 1H, H-2'), 4.20 (dd, J = 9.1, 7.7 Hz, 1H, H-2), 4.03 – 3.87 (m, 4H, H-4', H-6a, H-6b, H-6'a), 3.83 (ddd, J = 8.2, 5.4, 3.3 Hz, 1H, H-5'), 3.80 – 3.73 (m, 2H, H-6'b, H-4), 3.56 (s, 3H, OCH_3), 3.51 (ddd, J = 9.9, 4.9, 2.5 Hz, 1H, H-5); **^{13}C NMR** δ 101.50 (C-1, C-1'), 81.79 (C-3), 77.42 (C-3'), 77.38 (C-2), 77.21 (C-2'), 76.56 (C-4'), 75.56 (C-5'), 74.81 (C-5), 68.53 (C-4), 60.60 (C-6), 60.47 (C-6'), 56.98 (OCH_3); HRMS (ESI)

calculated for: $C_{13}H_{24}O_{23}S_4$ $[M–4H+2Na]^{2–}$ m/z 358.9541, found 358.9533; $C_{13}H_{24}O_{23}S_4$ $[M–4H+Na]^{3–}$ m/z 231.6395, found 231.6394; $C_{13}H_{24}O_{23}S_4$ $[M–4H]^{4–}$ m/z 167.9822, found 167.9821.

Supplementary Fig. 65 | ^{1}H-NMR spectrum of S14 (600 MHz, D_2O).

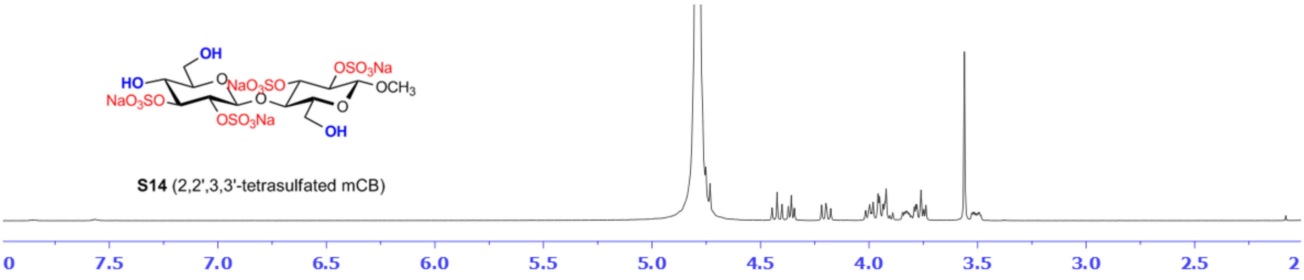

Supplementary Fig. 66 | ^{13}C-NMR spectrum of S14 (150 MHz, D_2O).

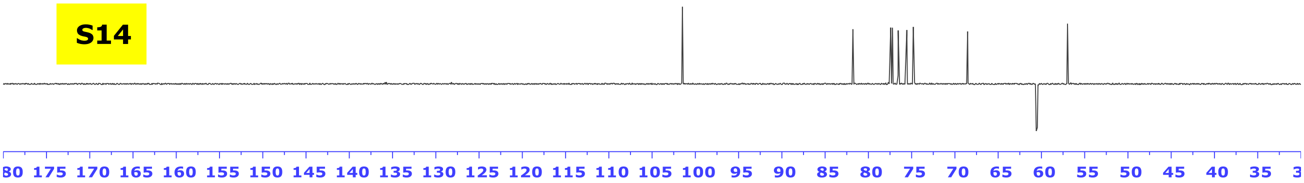

Supplementary Fig. 67 | 2D ^{1}H-^{1}H COSY and 2D ^{1}H-^{13}C HSQC spectrum of S14.

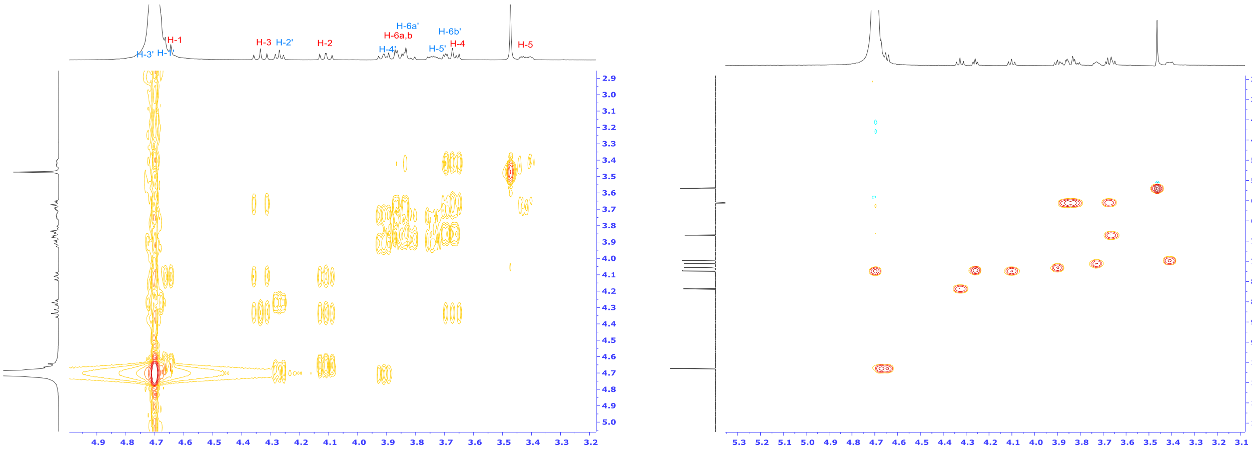

Supplementary Fig. 68 | HPLC analysis and HRMS spectrum of S14.

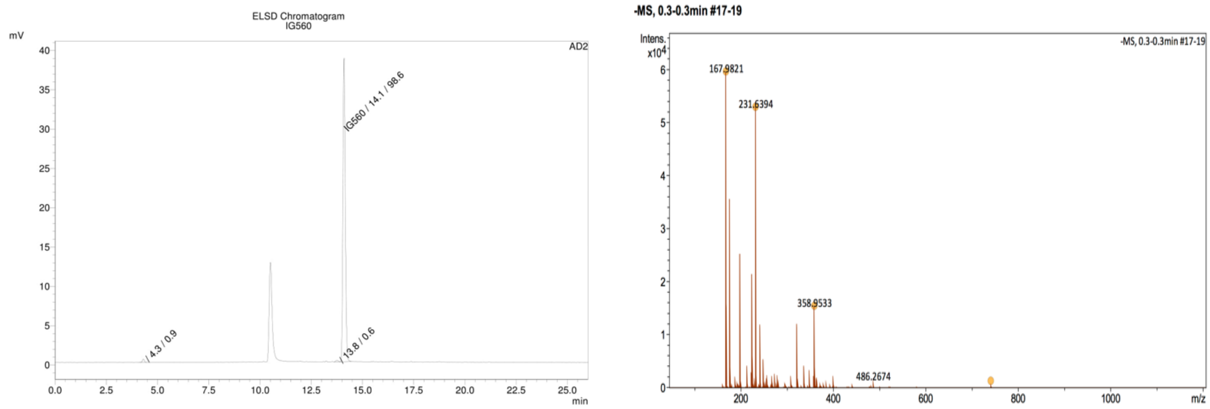

Methyl 2,3-di-*O*-sulfonato-β-D-glucopyranosyl-(1→4)-2,3,6-tri-*O*-sulfonato-β-D-glucopyranoside pentasodium salt (S15)

Compound **S15** was prepared from methyl β-D-cellobioside in 4% yield following procedures described in **Supplementary Scheme 4**. **^{1}H NMR** (600 MHz, D_2O) δ 4.80 – 4.77 (m, 2H, H-1, H-1'), 4.72 (appt, *J* = 6.9 Hz, 1H, H-3), 4.52 (dd, *J* = 11.0, 3.9 Hz, 1H, H-6a), 4.43 (appt, *J* = 9.1 Hz, 1H, H-3'), 4.38 (appt, *J* = 5.9 Hz, 1H, H-2), 4.31 (dd, *J* = 11.0, 2.7 Hz, 1H, H-6b), 4.21 (appt, *J* = 9.0 Hz, 1H, H-2'), 4.10 (appt, *J* = 8.1 Hz, 1H, H-4), 4.02 – 3.98 (m, 1H, H-5), 3.94 (dd, *J* = 12.6, 2.4 Hz, 1H, H-6'a), 3.80 – 3.74 (m, 2H, H-6'b, H-4'), 3.56 (s, 3H, OCH3), 3.52 (ddd, *J* = 9.6, 4.5, 2.2 Hz, 1H, H-5'); **^{13}C NMR** δ 100.97 (C-1), 100.80 (C-1'), 81.86 (C-3'), 77.68 (C-3), 77.66 (C-2'), 76.95 (C-2), 75.13 (C-4), 74.67 (C-5'), 72.66 (C-5), 68.59 (C-4'), 66.31 (C-6), 60.38 (C-6'), 56.88 (OCH_3); **HRMS** (ESI) calculated for: $C_{13}H_{24}O_{26}S_5$ $[M–5H+3Na]^{2–}$ m/z 409.9230, found 409.9218; $C_{13}H_{24}O_{26}S_5$ $[M–5H+2Na]^{3–}$ m/z 265.6188, found 265.6182; $C_{13}H_{24}O_{26}S_5$ $[M–5H+Na]^{4–}$ m/z 193.4666, found 193.4665.

Supplementary Fig. 69 | ^{1}H-NMR spectrum of S15 (600 MHz, D_2O).

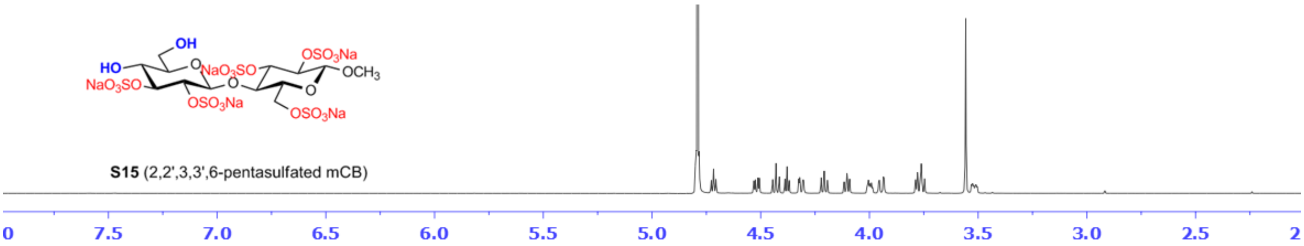

Supplementary Fig. 70 | ^{13}C-NMR spectrum of S15 (150 MHz, D_2O).

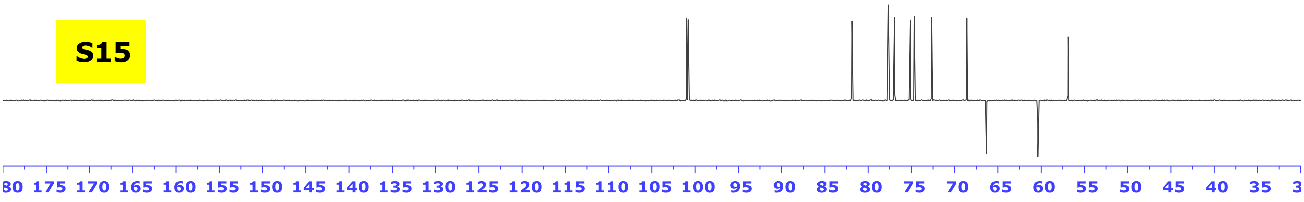

Supplementary Fig. 71 | 2D ^{1}H-^{1}H COSY and 2D ^{1}H-^{13}C HSQC spectrum of S15.

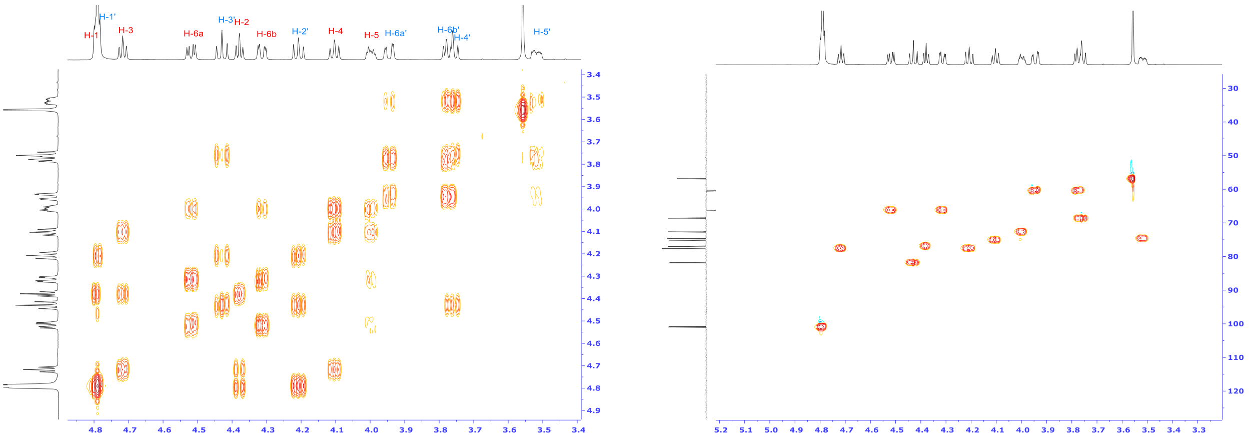

Supplementary Fig. 72 | HPLC analysis and HRMS spectrum of S15.

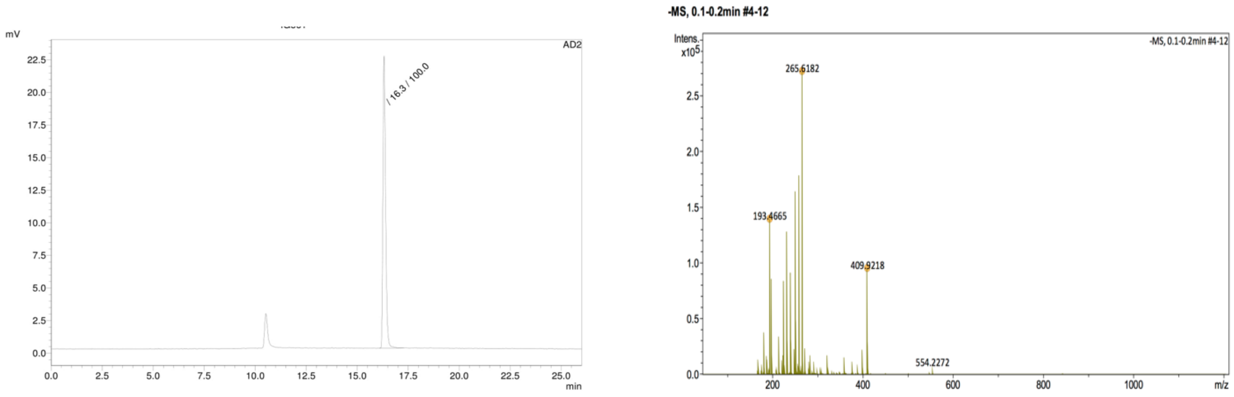

References

1. Wan, Y.; Zhang, X.-X.; Wang, C.; Zhao, L.-L.; Chen, L.-F.; Liu, G.-X.; Huang, S.-Y.; Yue, S.-N.; Zhang, W.-L.; Wu, H., *Tetrahedron* **2013,** *69* (19), 3947-3950.

2. Wessel, H. P.; Bartsch, S., *Carbohydr. Res.* **1995,** *274* (0), 1-9.

3. Probst, K. C.; Wessel, H. P., *J. Carbohydr. Chem.* **2001,** *20* (7-8), 549-560.

Properties of mCBS

Summary of Pharmacology and Toxicology of mCBS

Methyl β-D-cellobioside per-*O*-sulfate heptasodium, (or mCBS), is the heptasodium salt of methyl 2,2′,3,3′,4′,6,6′-hepta-*O*-sulfate-β-D-cellobioside (**S4**). It has been shown to be efficacious in various cell-based assays of extracellular histone-mediated toxicity.

mCBS is presented in 10 ml glass vials fitted with a rubber stopper and seal, as a sterile, concentrate solution (70 mg ml^{-1}) formulated in phosphate buffer. At this concentration the solution is isotonic (~300 mOsml kg^{-1}) with a pH of ~7.5. The drug product solution (labelled as STC3141) was diluted and prepared for dosing according to the clinical protocol, but is anticipated to be diluted to concentrations in the range of 1.5-28 mg ml^{-1} (mCBS heptasodium salt) using saline for injection, ready for intravenous (i.v.) infusion.

Nonclinical Pharmacology

The *in vitro* primary pharmacodynamic data indicates that mCBS exhibits the following properties:

- Protective against histone induced cell cytotoxicity in a concentration dependent manner and is able to reverse the damaging effect of histones
- Has a protective effect against histone-mediated RBC aggregation and able to inhibit histone-induced RBC aggregation dose dependently; also has a protective effect against histone-induced RBC lysis, able to attenuate histone-induced RBC fragility and reverse histone-mediated RBC toxicity
- Prevents histone-mediated platelet aggregation and degranulation in a dose-response manner
- Has minimal anticoagulant activity compared to low molecular weight heparin (LMWH) and unfractionated heparin (UFH)

Based on *in vitro* data, an effective concentration range for mCBS against histone-mediated toxic effects with histones at 400 μg ml^{-1}, which is close to the maximum concentration of circulating histones detected clinically, appears to be 50-200 μg ml^{-1}.

Two *in vivo* pharmacodynamic (PD) studies, one in the mouse and the other in the rabbit, respectively, showed that mCBS can mediate a reduction in histone-induced systemic toxicity. Single intraperitoneal (i.p.) doses of 6.25, 25 or 100 mg kg^{-1} (as sodium salt of mCBS) in the mouse showed a dose related reduction in cell injury induced by histones (50 mg kg^{-1}), as shown by a reduction in the biomarkers alanine aminotransferase (ALT), lactate dehydrogenase (LDH) and creatinine (Crea). The higher dose of mCBS, 100 mg kg^{-1}, showed near complete abolishment of the histone-induced increase in these biomarkers. An *in vivo* study in the rabbit showed i.v. administration of 50 or 100 mg kg^{-1} mCBS dose-proportionally decreased the levels of histone coated Tc-nanoparticles lodging in rabbit lung, thus indicating mCBS has the potential to reduce the accumulation of histones in the lung during disease, such as sepsis. Based on these *in vivo* PD data, the human equivalent dose (HED) of the pharmacologically active doses of mCBS, range from approximately 0.5 mg kg^{-1} in the mouse (i.p. route) up to 32 mg kg^{-1} in the rabbit (i.v. route) (expressed as sodium salt of mCBS).

The *in vitro* human Ether-a-go-go Related Gene (hERG) study indicated no potential liability for cardiac toxicity mediated via this mechanism at concentrations up to 2 mM. Lack of an effect on the cardiovascular system was also confirmed *in vivo* in the dog where i.v. infusion of doses up to 2785 mg kg^{-1} day^{-1} mCBS (free base) for 14 days had no effect on the morphology of the P-QRS-T wave complex or heart rate, PR, QRS duration, QT and QTc intervals. No effect on respiratory parameters (respiratory rate, tidal volume and minute volume) was seen following a single i.v. injection of mCBS in male rats at doses up to 848 mg kg^{-1} (free base). No effect on the central nervous system was indicated by detailed observation of rat behavior (general behavior, sensorimotor or autonomic) following 14 days continuous dosing (3000 mg kg^{-1} day^{-1})$^{.}$

Pharmacokinetics and metabolism in animals.

Overall, single dose pharmacokinetic (PK) parameters in rats indicated linearity between doses of 16.3 and 81.5 mg kg^{-1} (free base) given as a single i.v. bolus, with peak exposure at administration, a wide distribution through the body tissues (approximately 48 l kg^{-1}) and slow clearance (Cl) rates of approximately 0.6 l h^{-1} kg^{-1}. Around 50% of the dose was excreted intact in urine within 4 h post-dose and 69% within 48 h. Non-compartmental analysis of the plasma data showed that the elimination was biphasic, with most (>99%) of the material being eliminated within the first few hours with a half-life of 39 minutes and a long terminal elimination half-life of approximately 56 h. The data from the 7 days continuous i.v. infusion studies in the rat (n=3/sex) showed no sex differences, linearity for steady state concentrations and area under the concentration-time curve (AUC) 5-96 over the dose range 1394 to 5575 mg kg-^{1,} and Cl rates similar to a single dose (0.6-0.8

l h^{-1} kg $^{-1}$).

In the rat 14-day i.v. infusion study, over the dose range 300 to 3000 mg kg^{-1} day^{-1}, the mCBS plasma steady-state concentrations (C_{ss}) were reached within 5 h post start of infusion in all animals. The mean C_{ss} ranged from 20.9 to 242 µg ml^{-1}, while mean AUC_{0-336} (AUC_{0-360}) ranged from 6970 (7010) to 81000 (81400) µg h^{-1} ml^{-1}. After the end of infusion, the mean mCBS plasma concentrations declined rapidly at a mean estimated t. value ranging from 0.653 to 0.742 h and a mean Cl ranging from 0.47 to 0.6 l h^{-1} kg^{-1}. The mean volume of distribution (Vz) ranged from 481 to 590 ml kg^{-1}, suggesting that mCBS is largely distributed among tissues. The conclusion from this study was that AUC_{0-336}, AUC_{0-360} and C_{ss} values generally increased dose dependently and in a dose-proportional manner. There were no noteworthy sex-related differences for any of the measured toxicokinetic parameters.

In dogs, 48 h i.v. infusion over the dose range 348 to 2788 mg kg^{-1} day^{-1} showed near linear mean plasma steady-state concentrations (C_{ss}) of 53.8 to 358 µg ml^{-1} and clearance (Cl) at a rate of 0.22 to 0.45 l h^{-1} kg^{-1}. Pharmacokinetic parameters after a continuous i.v. infusion over a period of 6 days at a dose of 3840 mg kg^{-1} day^{-1} showed no sex differences, and rapid decline in mCBS plasma levels after dosing was stopped. The mean estimated initial t. value after repeat dosing was approximately 1 to 1.4 hour (initial elimination phase). This short half-life reflects the initial rapid elimination phase from plasma. The terminal phase half-life was not calculated in the dog. However, similar to the rat, mCBS was cleared in the dog at a mean rate of 0.2 to 0.5 l h^{-1} kg^{-1} and showed wide distribution into tissues.

In vitro, protein binding of mCBS in the human, rat and dog plasma was low (19-23%). Minimal *in vitro* metabolism was seen for mCBS in human, rat and dog liver microsomes under phase 1 or phase 2 conditions.

Toxicology.

Two Good Laboratory Practice (GLP) 14-day toxicity studies have been performed with mCBS administered by continuous i.v. infusion (24 h day$^{-1)}$ to rats and dogs.

The data from the 14-day rat toxicity study indicated a no-observed-adverse-effect level (NOAEL), following 14 days continuous i.v. infusion, of 300 mg kg^{-1} day^{-1}free base. At this level, mean (sexes combined) C_{ss} of 23.8 µg ml^{-1}, with an AUC_{0-360} of 7990 µg h^{-1} ml^{-1} was observed. Activated partial thromboplastin time (aPTT) was increased. The only notable macroscopic changes observed at 300

mg^{-1} kg^{-1} day^{-1} were enlarged bronchial and mediastinal lymph nodes, which was not observed in any of the animals after the 14-day recovery period. This dose was also associated with mild proximal tubular vacuolation/rarefaction in both kidneys which correlated with increased serum urea levels. There was also mild accumulation of foamy Kupffer cells in the liver and minimal to mild accumulation of foamy macrophages in the ovaries and uterus of female animals and in the adrenals and in various lymph nodes of all treated animals.

Mid and high doses of 1000 and 3000 mg kg^{-1} day^{-1} administered to the rat for 14 days (continuous infusion) showed no hematology changes at the end of infusion on day 15 but decreased red blood cell count (RBC), hemoglobin and hematocrit and an increase in reticulocytes (absolute and relative) at the end of the recovery phase in animals dosed with 3000 mg kg^{-1} day^{-1}. aPTT was increased in a dose-related manner and showed reversibility after a 14-day recovery period. Adverse findings in the kidneys were accompanied by increases in serum creatinine and urea. At these two doses, pale discoloration in the kidneys and enlargement in a number of lymph nodes as well as microscopic changes in the kidney, liver, ovaries, uterus, adrenals, spleen and lungs were observed, largely increasing in frequency and severity in a dose-dependent manner. Changes in the thymus and mammary glands were noted in a number of the high dose animals after 14 days infusion. After the 14-day recovery period in the high-dose treated rats, most of the microscopic changes recorded were still observed, however, the incidence and/or severity was decreased suggesting an ongoing recovery process. Test item-related changes were no longer observed in the lung, thymus and mammary glands of the high-dose recovery cohort.

The data from the 14-day GLP dog toxicity study indicated a NOAEL (278.5 mg kg^{-1} day^{-1}). At this level, mean (sexes combined) C_{ss} was observed at 49.85 µg ml^{-1}, with an $AUC_{0\text{-}360}$ of 16750 µg h^{-1} ml^{-1}. In the dog, 278.5 mg kg^{-1} day^{-1} produced transient increases in aPTT as well as mild microscopic changes consisting of minimal to mild vacuolation of the proximal tubules of the kidney, minimal diffuse cell infiltrate in the liver and minimal to mild foamy cell accumulation of the iliac lymph node. The microscopic changes in kidneys, liver and lymph nodes were mild in severity, and therefore considered to be non-adverse.

The continuous i.v. infusion (24 h day^{-1} for 14 days) of 849 and 2785 mg kg^{-1} day^{-1} mCBS (the mid and high study doses respectively) in the dog produced transient decreases in platelet counts and increase in aPTT, as well as minimal to mild microscopic changes consisting mainly of cortical vacuolation (adrenals), vacuolation of tunica muscularis in the digestive tract (stomach, duodenum, ileum, cecum and colon), vacuolation of the joint capsule cells (femur), vacuolation of proximal

tubules (kidneys) correlating with an increase in kidney weight, vacuolation of the submucosa (urinary bladder), diffuse cell infiltrate of the sinusoidal/portobiliary space (liver) correlating with an increase in liver weight and foamy cell accumulation of the lymph nodes (iliac, mandibular, mediastinal, mesenteric and pancreatic) and Peyer's patches (jejunum).

Additional changes seen at 2785 mg kg^{-1} day^{-1} mCBS included an increase in cholesterol, triglyceride, urea and creatinine serum levels. Microscopically, changes were similar to those observed at 849 mg kg^{-1} day^{-1} mCBS, but were observed at a slightly higher incidence and severity, and included also vacuolation of the tunica muscularis in the jejunum and rectum, vacuolation of the atrial myocardium (heart), as well as Kupffer cell hypertrophy in the liver.

At the end of the 14-day recovery period in dogs previously treated with 2785 mg kg^{-1} day^{-1}, all hematological, coagulation and clinical chemistry parameters had returned to baseline levels. All of the microscopic findings recorded in the study were still observed at a similar, or slightly lower incidence and severity after the recovery period, suggesting partial recovery and that a longer recovery period may be necessary before complete recovery is achieved. Kidney and liver weights were still increased after the 14-day recovery phase.

Based on the data above the pharmacology of the compound indicates prolongation of aPTT may be increased and platelet number reduced. Based on both the rat and dog data, the compound widely distributes into tissues. The vacuolation noted in tissues was widespread but generally minimal to mild in nature except for changes in the lymph nodes and femur which reached moderate and the kidney which reached severe. The kidney and liver also increased in weight and kidney was pale at necropsy. Thus, the main target organs appear to be the kidney and liver. Clinical protocols should include appropriate monitoring of hematology, coagulation, as well as include appropriate kidney and liver biomarkers.

The findings of accumulation of foamy macrophages (in the spleen and lymph nodes), foamy Kupffer cells (in the liver) and proximal tubular vacuolisation/rarefaction (in the kidneys) suggest an adaptive change of the mononuclear phagocytic system and kidneys in response to STC314, due to the phagocytosis and clearance of the test item and/or its degradation product(s). Other findings in the spleen were also considered to be an adaptive response to the activated phagocyte system.

mCBS did not cause mutations in the GLP *in vitro* Ames test or produce chromosome aberrations in human peripheral lymphocytes. mCBS did not induce any genotoxic activity in the *in vivo*

micronucleus assay performed in the continuous 14 day i.v. toxicology study in rats.

No studies investigating the effects of mCBS on fertility, fetal development or carcinogenicity have been performed in animals.

Anticoagulant Properties of mCBS

The anticoagulant activity of mCBS was investigated using rotational thromboelastometry (ROTEM) assays, these assays being performed by the Parish Laboratory, JCSMR. ROTEM analysis gives a holistic view of whole blood clotting as it provides information on the plasma coagulation cascade, the platelet contribution to clot formation as well as the rate of clot lysis. Clotting Time (CT) represents the time it takes from initiation of the clotting assay to the point at which a clot of 2 mm in size has formed and essentially represents the period from the activation of the plasma coagulation cascade to the point at which fibrin is formed. The subsequent contribution of platelets and fibrin can be determined by the amplitude of the clot, or size in mm, at various time points including 5, 10 and 20 min following the end of the CT.

The ROTEM assay can be manipulated to provide more specific information on the contribution of the extrinsic and intrinsic pathways of coagulation as well as the contribution of fibrin formation through the use of specific reagents added to the blood to initiate the assay. The type of reagent added determines the name of the assay hence the 'NATEM' assay is the non-activated assay; the 'EXTEM' is specific for the extrinsic pathway of coagulation; the 'INTEM' is specific for the intrinsic pathway of coagulation and the 'FIBTEM' is specific for the contribution of fibrin to clot formation.

The effect of mCBS on clotting parameters using the different ROTEM assays was investigated, with whole blood being supplemented with mCBS (200 $\mu g\ ml^{-1}$) immediately prior to initiation of coagulation and the results being shown in Supplementary Fig. 9. It was found that mCBS, at a concentration (200 $\mu g\ ml^{-1}$) that would be rarely if ever reached in patients, did not activate the extrinsic pathway of coagulation (EXTEM assay). This was an interesting result as it implies that the extrinsic pathway of coagulation that is triggered by tissue factor was not altered by the addition of mCBS. Furthermore, mCBS did not aid the contribution of fibrin to clot formation (FIBTEM assay) and only very weakly activated the intrinsic pathway (INTEM assay). In contrast, mCBS did exhibit some anticoagulant activity in the NATEM assay that measures coagulation in the absence of exogenous activators (Supplementary Fig. 9).

Comparison of the anticoagulant activity of mCBS with LMWH and UFH.

Whole blood was supplemented with a range of concentrations of mCBS, LMWH or UFH (200 – 0.2 μg ml^{-1}) immediately prior to the NATEM assay being undertaken. Compounds were compared at each concentration within assays that included a control consisting of an equivalent volume of deionized water. Data represent clotting time expressed as a percentage of the water control (Supplementary Fig. 10a). This analysis demonstrated that mCBS at 200 μg ml^{-1} had a modest impact upon whole blood coagulation whereas LMWH at 10%, and UFH at 1% of the mCBS concentration prevented clot formation. Comparison of the 3 compounds at concentrations that would permit clot formation to be measured was able to be carried out when LMWH and UFH concentrations were lowered 100- and 1000-fold, respectively, relative to mCBS (Supplementary Fig. 10b). At these concentrations, LMWH and UFH showed clotting times of 1.1x and 0.764x that of mCBS, respectively. Thus, taking into account the 100- and 1000-fold dilutions, the results demonstrated that the anticoagulant activity of mCBS in the NATEM assay is 110 times lower than LMWH and 764 times lower than UFH. Of course, this analysis probably gives an inflated estimate of the anti-coagulant activity of mCBS as the compound gave little or no activity in the EXTEM, INTEM and FIBTEM assays (Supplementary Fig. 9).

Chemical Stability of CBS and mCBS

Stability studies were carried out at 5±3 °C, 25±2 °C and 40±2 °C, with levels of both mCBS and cellobiose sulfate (CBS) being tracked in formulated clinical material (i.e., mCBS in phosphate buffer, pH 7.5) via HPLC. Graphing the percent (%) change of mCBS and CBS relative to their starting amount (at T=0) revealed that mCBS levels remained unchanged after incubation at 5 °C and 25 °C for 25 months and 40 °C for 13 months, indicating that mCBS is a very stable compound, even when incubated in aqueous solution at 40 °C for 13 months (Supplementary Fig. 3a-c). In contrast, CBS levels declined rapidly and in a temperature dependent manner. Thus, at 5 °C approximately 50% of CBS had decomposed after 1 month and over 90% at 6-13 months (Supplementary Fig. 3a), whereas at 25 °C and 40 °C essentially 100% of CBS had decomposed following storage in aqueous solution for only 1 month (Supplementary Fig. 3b,c). These data indicate that CBS is highly unstable in aqueous solutions but the addition of a methyl group to the reducing terminus of CBS results in a molecule (mCBS) that is very stable when stored under aqueous conditions at a range of temperatures. On the other hand, when stored as a powder at -20 °C for 25 months, both mCBS and CBS were found to be highly stable.

In additional studies, mCBS stability was assessed when formulated in different buffered aqueous solutions other than PBS, namely citrate and acetate buffered saline (pH 7.5). The data from these experiments revealed that the high stability of mCBS in PBS at 5 °C, 25 °C and 40 °C was recapitulated when mCBS was stored in citrate and acetate buffered solutions at pH 7.5.
