# Supplementary material for: Neutralizing the pathological effects of extracellular histones with small polyanions
Source: Nat Commun. 2020 Dec 16;11:6408. doi: 10.1038/s41467-020-20231-y (PMC7744542; doi:10.1038/s41467-020-20231-y)
Supplement: Supplementary file 3 — Reporting Summary [file 41467_2020_20231_MOESM3_ESM.pdf]

nature research

Corresponding author(s): Christopher R Parish
Last updated by author(s): Oct 28, 2020

Reporting Summary

Nature Research wishes to improve the reproducibility of the work that we publish. This form provides structure for consistency and transparency in reporting. For further information on Nature Research policies, see Authors & Referees and the Editorial Policy Checklist .

Statistics

For all statistical analyses, confirm that the following items are present in the figure legend, table legend, main text, or Methods section.

n/a	Confirmed	
☐	☒	The exact sample size (*n*) for each experimental group/condition, given as a discrete number and unit of measurement
☐	☒	A statement on whether measurements were taken from distinct samples or whether the same sample was measured repeatedly
☐	☒	The statistical test(s) used AND whether they are one- or two-sided<br>*Only common tests should be described solely by name; describe more complex techniques in the Methods section.*
☐	☒	A description of all covariates tested
☐	☒	A description of any assumptions or corrections, such as tests of normality and adjustment for multiple comparisons
☐	☒	A full description of the statistical parameters including central tendency (e.g. means) or other basic estimates (e.g. regression coefficient) AND variation (e.g. standard deviation) or associated estimates of uncertainty (e.g. confidence intervals)
☐	☒	For null hypothesis testing, the test statistic (e.g. *F*, *t*, *r*) with confidence intervals, effect sizes, degrees of freedom and *P* value noted<br>*Give P values as exact values whenever suitable.*
☒	☐	For Bayesian analysis, information on the choice of priors and Markov chain Monte Carlo settings
☒	☐	For hierarchical and complex designs, identification of the appropriate level for tests and full reporting of outcomes
☒	☐	Estimates of effect sizes (e.g. Cohen's *d*, Pearson's *r*), indicating how they were calculated

Our web collection on statistics for biologists contains articles on many of the points above.

Software and code

Policy information about availability of computer code

Data collection	Microsoft Excel 2019 v16.31., FACSDIVA v 8.0.1, Flowjo v10.7.1., CellQuest Pro Software v 5.2.1.
Data analysis	Microsoft Excel 2019 v16.31., Graphpad Prism v 8.4.3., Flowjo v 10.7.1., Biorad ChemiDoc Imaging System, Image lab, v 5, Fiji ImageJ v2.

For manuscripts utilizing custom algorithms or software that are central to the research but not yet described in published literature, software must be made available to editors/reviewers. We strongly encourage code deposition in a community repository (e.g. GitHub). See the Nature Research guidelines for submitting code & software for further information.

Data

Policy information about availability of data

All manuscripts must include a data availability statement. This statement should provide the following information, where applicable:

- Accession codes, unique identifiers, or web links for publicly available datasets
- A list of figures that have associated raw data
- A description of any restrictions on data availability

The authors declare that the data supporting the findings of this study are available within the paper and its supplementary information files. Associated raw data available for Figs.1-8, Supp Figs 1, 2, 4-7, 9 and 10.

Field-specific reporting

Please select the one below that is the best fit for your research. If you are not sure, read the appropriate sections before making your selection.

☒ Life sciences ☐ Behavioural & social sciences ☐ Ecological, evolutionary & environmental sciences

For a reference copy of the document with all sections, see nature.com/documents/nr-reporting-summary-flat.pdf

Life sciences study design

All studies must disclose on these points even when the disclosure is negative.

Sample size

To determine the number of experimental replicates (n) required to statistically validate any difference observed, we have used the following formula, n= 1 + 2C(s/d)x(s/d); where c is a constant that has a value dependent on our threshold of accepting false negatives (0.05 in our case) and false positives (0.2 or 0.1 in our case) and thus giving a value of C=7.85 or 10.51 (from statistical tables; not show). s refers to the standard deviations observed (we have used outcomes of experiments we have previously performed that are similar to those proposed experiment to estimate this) and d refers to the difference observed (typically we have set this at 4-5%; that is, we hope to statistically validate 4-5% differences observed in our continuous variable data). From this analysis, we anticipate n=3-5 to statistically validate most experimental data (both in vitro and in vivo) to a resolution of 4-5% change in observed experimental outcomes. Typically, this will be applied based on the assumptions of the data being normally distributed and analysis being between two group means of continuous variables. It may be that the normal distribution of data assumption will be met by transforming the data through log transformations. ANOVA and and post ANOVA multicomparisons (with correction) will be used to test the data that adhere to the assumptions; otherwise non parametric (typically) rank sum-type analysis will be employed. We routinely ask for advice from the statistical consulting unit at our institute.

Data exclusions

There was no data exclusion in the study.

Replication

The number of replicates are indicated as exact numbers in the figure legends. The pathology experiments were replicated 3-9 times in independent experiments in various iterations with each experiment typically having 3 animals per group.

Randomization

Experiments treatments were spread across mice housed in each cage effectively randomizing groups throughout the entire sample population. All in vitro studies were performed in 96 well plates and, to avoid plate position effects, the position of groups within plates was randomly varied. Controls were also placed frequently and randomly within plates.

Blinding

For pathology data, plasma biochemistry was performed in an independent laboratory with samples assigned non-identifying unique codes, thus blinding the biochemistry analysis. For in vitro studies treatment groups were coded and the code broken after completion of data collection.

Reporting for specific materials, systems and methods

We require information from authors about some types of materials, experimental systems and methods used in many studies. Here, indicate whether each material, system or method listed is relevant to your study. If you are not sure if a list item applies to your research, read the appropriate section before selecting a response.

Materials & experimental systems

n/a	Involved in the study	
☐	☒	Antibodies
☐	☒	Eukaryotic cell lines
☒	☐	Palaeontology
☐	☒	Animals and other organisms
☐	☒	Human research participants
☒	☐	Clinical data

Methods

n/a	Involved in the study	
☒	☐	ChIP-seq
☐	☒	Flow cytometry
☒	☐	MRI-based neuroimaging

Antibodies

Antibodies used

Antibodies used were a mouse monoclonal anti-heparan sulfate (HS) antibody (Amsbio, clone 10E4, IgM isotype), rabbit polyclonal anti-histone 3 and anti-histone 4 antibodies (BioVision, cat. no. 3623 and 3624, respectively) and a secondary polyclonal goat F(ab')2 anti-mouse IgM-PE antibody (SouthenBiotech, cat. no. 1022-09) to detect binding of the anti-HS-specific antibody to cells.

Validation

The primary anti-HS mAb was validated by failure to react with the HS-deficient cell line, pgsA-745, but still able to react strongly with the parent CHO-K1 cell line. In the case of the anti-histone 3 and anti-histone 4 primary antibodies we relied on the target validation stated by the manufacturer i.e., in western blots specifically detect human histone 3 or human histone 4 at appropriate bandsize.

Eukaryotic cell lines

Policy information about cell lines

Cell line source(s)

Human umbilical vein endothelial cells (HUVEC) were established from primary cultures as previously described and monitored for their characteristic cobble stone appearance when grown as confluent monolayers. Human microvascular endothelial cells-1 (HMEC-1) and Chinese Hamster Ovary (CHO)-K1 cells and xylotransferase-1-deficient CHO-K1 cells (pgsA-745 cells) that are HS and GAG deficient, were supplied by the ATCC.

Authentication

Primary cultures of HUVEC were identified by their cobblestone appearance and were discarded after 6-8 passages. HMEC-1 were continually tested for their characteristic resistance to histone cytotoxicity. The CHO-K1 cell lines were monitored for presence (CHO-K1 cell line) or absence (CHO-KI pgsA-745 cell line) of cell surface heparan sulfate by flow cytometry using a

	HS-specific mAb.
Mycoplasma contamination	Cell lines were used that tested negative for mycoplasma contamination
Commonly misidentified lines (See ICLAC register)	No commonly misidentified cell lines were used in this study

Animals and other organisms

Policy information about studies involving animals; ARRIVE guidelines recommended for reporting animal research

Laboratory animals	Pathogen free male and female C57BL/6 mice (6-8 weeks of age), female BALB/c mice (5-6 weeks of age) and male Wistar rats (8-12 weeks of age and weighing between 250-350g) were used.
Wild animals	The study did not involve wild animals
Field-collected samples	The study did not involve specimens collected from the field.
Ethics oversight	All animal experiments were approved by the Australian National University Animal Experimentation Ethics Committee.

Note that full information on the approval of the study protocol must also be provided in the manuscript.

Human research participants

Policy information about studies involving human research participants

Population characteristics	Healthy adult donors were used as a source of erythrocytes and platelets for in vitro studies. Consenting patients admitted to The Canberra Hospital Intensive Care Unit with an APACHE II mortality score ≥12 on arrival in the ICU and a diagnosis of sepsis were included in our study. No patient information was provided except that they were adults and sepsis patients based on an APACHE II mortality score of >12.
Recruitment	Healthy adult donors were volunteers from research labs at the John Curtin School of Medical Research. To avoid bias multiple blood collections from the same individual was avoided. Sepsis patients were identified upon arrival at the ICU, The Canberra Hospital, based on an APACHE II mortality score of ≥12. Blood samples were collected from consenting sepsis patients as soon as possible after diagnosis to avoid subsequent treatments interfering with plasma NET levels.
Ethics oversight	All human-related research was approved by the ACT Health Human Research Ethics Committee.

Note that full information on the approval of the study protocol must also be provided in the manuscript.

Flow Cytometry

Plots

Confirm that:

- [x] The axis labels state the marker and fluorochrome used (e.g. CD4-FITC).
- [x] The axis scales are clearly visible. Include numbers along axes only for bottom left plot of group (a 'group' is an analysis of identical markers).
- [x] All plots are contour plots with outliers or pseudocolor plots.
- [x] A numerical value for number of cells or percentage (with statistics) is provided.

Methodology

Sample preparation	All details of sample preparation are outlined in the methods.
Instrument	BD flow cytometers were used throughout the study and included the FACSCalibur, LSR-II, LSRFortessa and LSRFortessa X-20 models models.
Software	FACSDIVA and Flowjo software were used to collect and/or analyze the data.
Cell population abundance	Sample analysis typically involved 10,000 events or more of the target population.
Gating strategy	Gating strategies are clearly shown in the figures and/or associated supplementary figures and typically involved gating out debris and/or gating on target populations based on FSC and SSC.

- [x] Tick this box to confirm that a figure exemplifying the gating strategy is provided in the Supplementary Information.
